# Prognostic, predictive, and pharmacogenomic assessments of CDX2 refine stratification of colorectal cancer

**DOI:** 10.1002/1878-0261.12347

**Published:** 2018-08-15

**Authors:** Jarle Bruun, Anita Sveen, Rita Barros, Peter W. Eide, Ina Eilertsen, Matthias Kolberg, Teijo Pellinen, Leonor David, Aud Svindland, Olli Kallioniemi, Marianne G. Guren, Arild Nesbakken, Raquel Almeida, Ragnhild A. Lothe

**Affiliations:** ^1^ Department of Molecular Oncology Institute for Cancer Research the Norwegian Radium Hospital Oslo University Hospital Norway; ^2^ K.G. Jebsen Colorectal Cancer Research Centre Clinic for Cancer Medicine Oslo University Hospital Norway; ^3^ Institute of Molecular Pathology and Immunology University of Porto (IPATIMUP) Portugal; ^4^ Instituto de Investigação e InovaçãoemSaúde (i3S) Porto Portugal; ^5^ Faculty of Medicine University of Porto Portugal; ^6^ Institute for Molecular Medicine Finland (FIMM) University of Helsinki Finland; ^7^ Institute for Clinical Medicine Faculty of Medicine University of Oslo Norway; ^8^ Department of Pathology Oslo University Hospital Norway; ^9^ Science for Life Laboratory Solna Sweden; ^10^ Department of Oncology and Pathology Karolinska Institutet Solna Sweden; ^11^ Department of Oncology Oslo University Hospital Norway; ^12^ Department of Gastrointestinal Surgery Aker Hospital – Oslo University Hospital Norway; ^13^ Biology Department Faculty of Sciences University of Porto Portugal

**Keywords:** CDX2, colorectal cancer, drug sensitivity, pharmacogenomics, predictive biomarker, prognostic biomarker

## Abstract

We aimed to refine the value of CDX2 as an independent prognostic and predictive biomarker in colorectal cancer (CRC) according to disease stage and chemotherapy sensitivity in preclinical models. CDX2 expression was evaluated in 1045 stage I–IV primary CRCs by gene expression (*n *=* *403) or immunohistochemistry (*n *=* *642) and in relation to 5‐year relapse‐free survival (RFS), overall survival (OS), and chemotherapy. Pharmacogenomic associations between CDX2 expression and 69 chemotherapeutics were assessed by drug screening of 35 CRC cell lines. CDX2 expression was lost in 11.6% of cases and showed independent poor prognostic value in multivariable models. For individual stages, CDX2 was prognostic only in stage IV, independent of chemotherapy. Among stage I–III patients not treated in an adjuvant setting, CDX2 loss was associated with a particularly poor survival in the *BRAF*‐mutated subgroup, but prognostic value was independent of microsatellite instability status and the consensus molecular subtypes. In stage III, the 5‐year RFS rate was higher among patients with loss of CDX2 who received adjuvant chemotherapy than among patients who did not. The CDX2‐negative cell lines were significantly more sensitive to chemotherapeutics than CDX2‐positive cells, and the multidrug resistance genes *MDR1* and *CFTR* were significantly downregulated both in CDX2‐negative cells and in patient tumors. Loss of CDX2 in CRC is an adverse prognostic biomarker only in stage IV disease and appears to be associated with benefit from adjuvant chemotherapy in stage III. Early‐stage patients not qualifying for chemotherapy might be reconsidered for such treatment if their tumor has loss of CDX2 and mutated *BRAF*.

**Abbreviations**
5FU: 5‐fluorouracil
BASC: Binarization Across Multiple Scales
CDX2: Caudal type homeobox 2 transcription factor
CI: confidence interval
CMS: consensus molecular subtypes
CRC: colorectal cancer
DSRT: drug sensitivity and resistance testing
DSS: drug sensitivity score
FBS: fetal bovine serum
G1: high differentiation
G2: moderate differentiation
G3: poor differentiation
GEO: Gene Expression Omnibus
GSA: gene set analysis
HR: hazard ratio
MSI: microsatellite instability
MSS: microsatellite‐stable
ND: not determined
OS: overall survival
pCRC: primary colorectal cancer
RFS: relapse‐free survival
TMA: tissue microarray
TNM: tumor node metastasis

## 1. Introduction

Worldwide, 1.4 million patients are diagnosed with colorectal cancer (CRC) each year, and the five‐year mortality rate is about 50% (Torre *et al*., 2015). The tumor node metastasis (TNM) classification system provides the main clinical framework to assess CRC prognosis, and combined with clinicopathological characteristics and a few molecular markers, it forms the conventional basis to estimate prognosis and guide adjuvant treatment decisions. As a significant percentage of patients with stage II and III CRC have a good prognosis, the risks of chemotherapy must be outweighed by the survival benefits. Prognosis differs significantly within clinically relevant subgroups, and to discover robust biomarkers that identify patients with a high risk of relapse who will benefit from adjuvant chemotherapy remains a major challenge.

The hypermutator phenotype microsatellite instability (MSI) accounts for 15% of primary CRCs, and patients with sporadic MSI tumors have a good prognosis (Lothe *et al*., 1993; Popat *et al*., 2005). MSI is recommended for clinical use as a low‐risk marker in patients with stage II colon cancer (Duffy *et al*., 2014; Merok *et al*., 2013), in particular as MSI tumors respond poorly to adjuvant 5‐fluorouracil (5FU) (Sargent *et al*., 2010). In 2017, MSI was also approved by the FDA as the first pan‐cancer biomarker for prediction of response to immune checkpoint inhibition in stage IV disease.

Recently, four gene expression‐based consensus molecular subtypes (CMS) of CRC were defined (Guinney *et al*., 2015). This classification has prognostic value independent of cancer stage, recognizing a mesenchymal subtype (CMS4) associated with poor prognosis and poor response to standard oncological treatment with chemotherapy (Song *et al*., 2016; Trinh *et al*., 2017). Currently, however, clinical use of gene expression‐based subtypes is limited, awaiting development of clinically useful assays. In contrast, molecular pathology provides a clinically feasible diagnostic toolbox and promises to deliver more accurate prognostics and response prediction. Several biomarkers have shown promise in CRC (Birgisson *et al*., 2010; Bruun *et al*., 2015; Schetter *et al*., 2008), but very few have been validated in large independent datasets, except from the Immunoscore (El Sissy *et al*., 2017; Galon *et al*., 2006), which is a prognostic tool that quantifies the levels of CD3‐ and CD8‐positive cells in the tumor center and at the invasive margin.

The caudal type homeobox 2 transcription factor (CDX2) is an emerging biomarker in CRC (Dalerba *et al*., 2016) and is currently used in the clinic for diagnosis of intestinal adenocarcinomas as it is a relatively sensitive and specific intestinal marker. CDX2 is also a particularly useful biomarker to classify cancers of unknown origin, when used together with other markers in a panel. This transcription factor is a major regulator of intestinal development and differentiation (Verzi *et al*., 2011), and it is specifically expressed in the intestinal epithelium (Werling *et al*., 2003). CDX2 is a tumor suppressor in the adult colon, and loss of CDX2 expression is associated with advanced stages of CRC, poor differentiation, *BRAF* mutation, and MSI (Olsen *et al*., 2014), as well as the CMS1 and CMS4 subtypes (Pilati *et al*., 2017; Trinh *et al*., 2017). In concordance, loss of CDX2 expression has been found to be associated with a poor patient prognosis in several studies (Baba *et al*., 2009; Dalerba *et al*., 2016; Lugli *et al*., 2008; Zhang *et al*., 2017), and it was recently suggested that the prognostic value is limited to the CMS4 group (Pilati *et al*., 2017). In a landmark study, CDX2 was proposed to have both prognostic and predictive value for benefit from chemotherapy in both stage II and stage III, separately (Dalerba *et al*., 2016). However, studies are needed to assess its prognostic value within individual cancer stages, while controlling for the impact of the most clinically relevant parameters known to be associated with CDX2 expression.

We aimed to determine the stage‐specific prognostic and predictive value of CDX2 by gene expression and *in situ* protein expression analyses of two population‐representative Norwegian series, relative to relevant clinical and molecular markers. We further explored the association between CDX2 expression and sensitivity to 69 conventional chemotherapeutics by pharmacogenomic profiling of 35 CRC cell lines.

## 2. Methods

This manuscript was based on the REMARK guidelines for reporting of biomarker studies (McShane *et al*., 2005) (Table S1).

### 2.1. Patient samples

**Figure 1:**
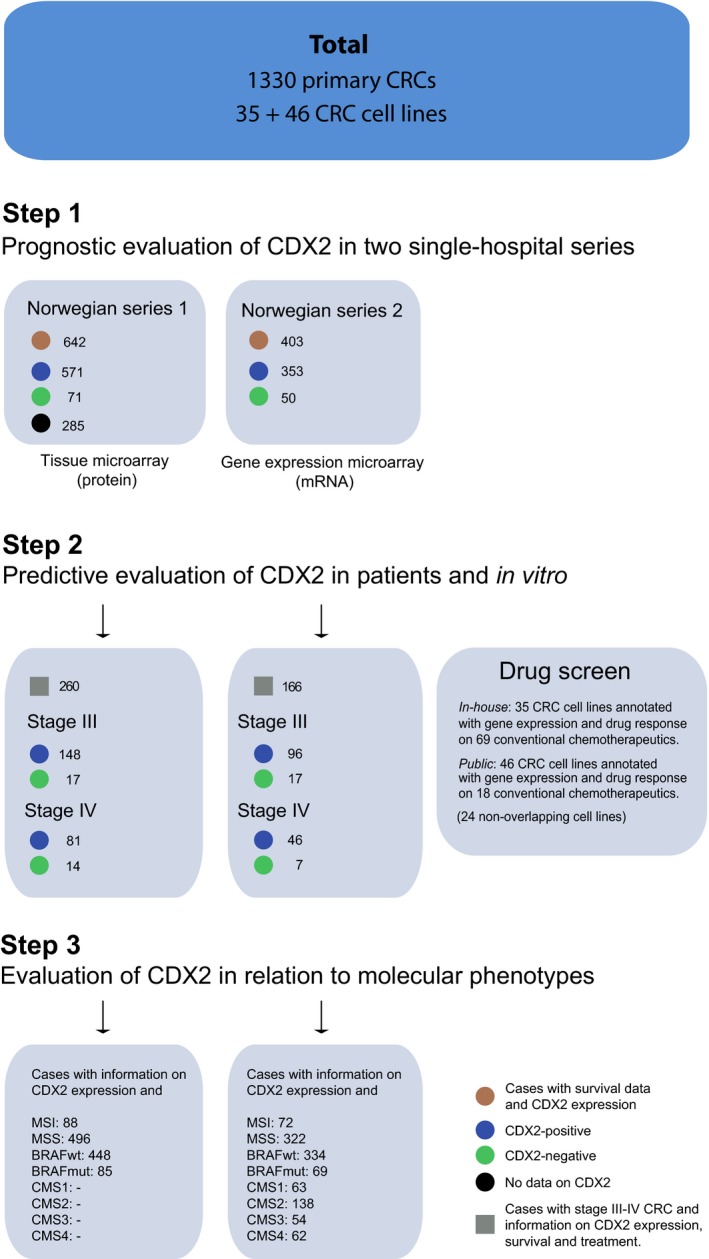
Study outline—patients and cell lines included in the study. Two Norwegian series were used to retrospectively assess the prognostic and predictive value of CDX2 expression in totally 1330 patients diagnosed with primary colorectal cancer (CRC), where 1045 were scored for CDX2 expression. Associations with clinically relevant molecular markers (microsatellite instability, *BRAF*‐mutation status, and consensus molecular subtype) were subsequently determined. Abbreviations: CMS, consensus molecular subtype; MSI, microsatellite‐instable; MSS, microsatellite‐stable.

Two independent single‐hospital patient series of primary CRC were analyzed for CDX2 expression (Fig. 1). Patients in the Norwegian series 1 (*n *=* *927) and the Norwegian series 2 (*n *=* *403) underwent major resection surgery at Oslo University Hospital, Aker, in the time periods 1993 to 2003 and 2006 to 2013, respectively (additional details in Table S2). This hospital serves a geographically defined catchment area with a population of about 270 000 inhabitants. All relevant clinical data have been recorded in a local database and quality‐controlled at follow‐ups. Data on all patients diagnosed with CRC are recorded in the Cancer Registry of Norway, and our data were cross‐checked with this database. The series are population‐representative for the Oslo area, and of adequate size to perform relevant subgroup analyses. Information on tumor location, histopathological grade, stage, and chemotherapy was registered. Data were collected prospectively and analyzed retrospectively. For the Norwegian series 1, DNA was extracted, MSI status was determined, and a tissue microarray (TMA) was built from matching formalin‐fixed paraffin‐embedded tumor tissue, as previously described (Bruun *et al*., 2015; Merok *et al*., 2013). For the Norwegian series 2, DNA and RNA were extracted from fresh‐frozen tumor tissue and MSI status was determined, as previously described (Berg *et al*., 2010). Sequencing of *BRAF* in exon 15 (including codon 600) was performed on a 3730 DNA Analyzer (Applied Biosystems, Foster City, CA, USA) as previously described (Berg *et al*., 2010). The two series were merged to increase the statistical power of the various subgroup analyses as the separate results were highly comparable (Table S2 and Figs S2 and S3). Furthermore, CDX2 gene and protein expression have been shown to correlate strongly in tissue (Olsen *et al*., 2016), and we found that they also correlate strongly in cell lines (Pearson correlation *r *=* *0.87, *n *=* *29; Fig. S1).

This project was endorsed by the Norwegian Data Protection Authority and the Regional Committee for Medical and Health Research Ethics, South‐Eastern Norway (REK number 1.2005.1629), and informed consent was obtained from all patients prior to enrollment. The research was carried out according to the Declaration of Helsinki, and the research biobanks were constructed according to national legislation.

### 2.2. Gene expression analyses

Gene expression data from the Norwegian series 2 (*n *=* *403; Table S2) were generated with exon‐level microarrays using two platforms: the Affymetrix Human Exon 1.0 ST (HuEx‐1_0‐st‐v2; *n *=* *236) or Human Transcriptome 2.0 Arrays (HTA 2.0; *n *=* *167; Affymetrix Inc., Santa Clara, CA, USA). Raw intensity data for samples analyzed on the Human Exon Array were background‐corrected, quantile‐normalized, and summarized at the gene level according to the robust multi‐array average (RMA) (Irizarry *et al*., 2003) approach implemented in the affymetrix expression console 1.1 software. Samples analyzed on the Human Transcriptome Array were preprocessed according to the modified Signal Space Transformation algorithm of RMA (Affymetrix). Gene expression data generated from the two different platforms were matched by HUGO gene symbols and merged by batch correction using ComBat (Johnson *et al*., 2007) implemented in the R library SVA. Gene expression values for CDX2 were retrieved from probe set ID 3507134 for the Human Exon Array and probe set ID TC13000513.hg.1 for the Human Transcriptome Array. The data have partly been published (*n *=* *236; NCBI's Gene Expression Omnibus (GEO) accession numbers http://www.ncbi.nlm.nih.gov/geo/query/acc.cgi?acc=GSE24550, http://www.ncbi.nlm.nih.gov/geo/query/acc.cgi?acc=GSE29638, http://www.ncbi.nlm.nih.gov/geo/query/acc.cgi?acc=GSE69182, and http://www.ncbi.nlm.nih.gov/geo/query/acc.cgi?acc=GSE79959), and the remaining data will be published elsewhere (Sveen *et al*., manuscript). The samples were classified according to CMS using the random forest predictor implemented in the R library CMSclassifier with a default posterior probability of 0.5 (Guinney *et al*., 2015).

### 2.3. Immunohistochemistry

The *in situ* nuclear protein expression of CDX2 was analyzed on a TMA by immunohistochemistry following standard protocols on 4‐μm‐thick tissue sections. Briefly, following deparaffinization in xylene and rehydration in graded ethanols, antigen retrieval was performed in a IHC‐Tek Epitope Retrieval Steamer Set (IHC World, Woodstock, MD, USA), for 40 min with 10 mm citrate buffer, pH 6.0. Endogenous peroxidase was blocked with 3% hydrogen peroxide in distilled water for 10 min. Incubation with primary antibody against CDX2 (1 : 50 dilution, mouse monoclonal CDX2‐88 clone of the IgG1 isotype; Biogenex, San Ramon, CA, USA) was performed overnight at 4°C. Sections were then incubated with the Dako REAL™ Envision™ Detection System Peroxidase/DAB+ (DAKO, Glostrup, Denmark) according to the manufacturer's instructions, for staining detection. Tissue sections were counterstained with Gill's hematoxylin (Leica Microsystems, Amersham, Bucks, UK), dehydrated in graded ethanols, clarified with xylene, and mounted using a xylene‐compatible mounting medium (Thermo Fisher Scientific, Cheshire, UK). Normal colonic mucosa served as a positive control for CDX2 expression. The primary antibody was omitted from one slide to provide a negative control.

Thirty‐one percent of the cases on the TMA were not evaluable with regard to CDX2 protein expression, largely due to tissue loss after repeated sectioning of the TMA, but some spots had poor tumor preservation, insufficient number of epithelial tumor cells, or extensive necrosis. There was no significant difference in patient characteristics between evaluable and unevaluable cases, suggesting that cases with CDX2 protein expression are representative of the full series (Table S3).

### 2.4. Dichotomization of CDX2 gene and protein expression

**Figure 2:**
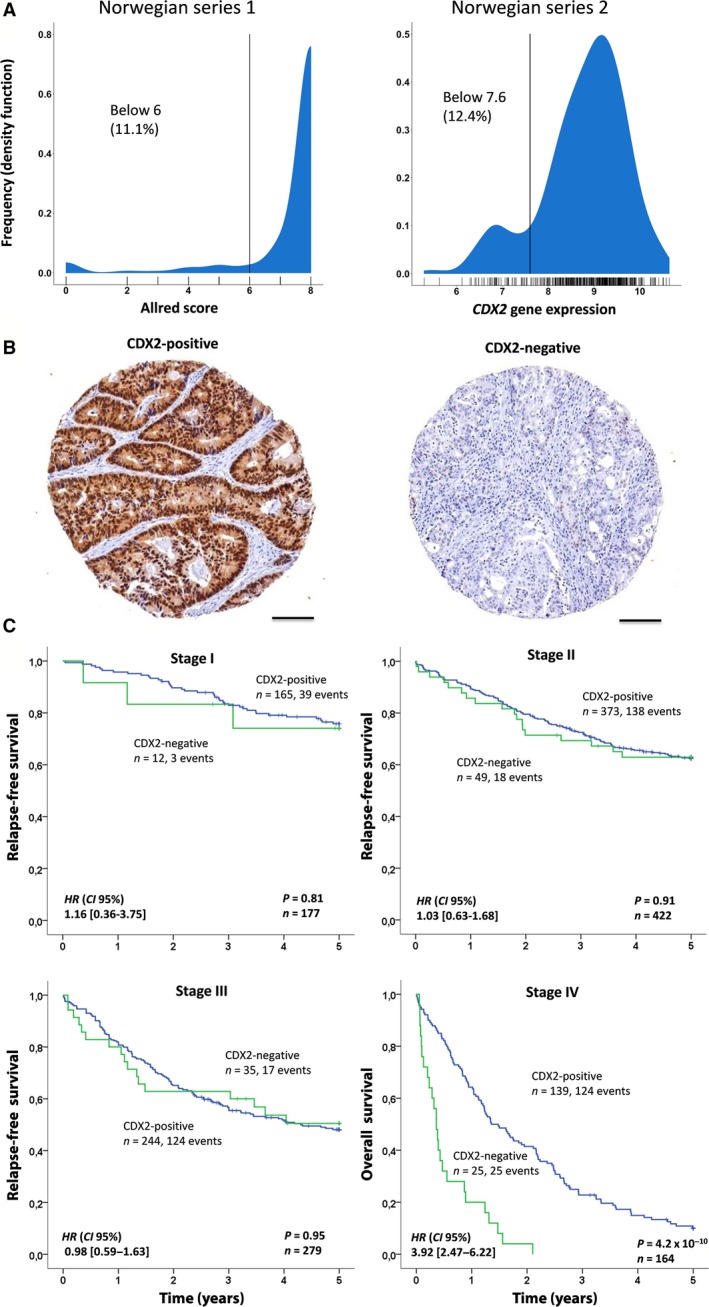
Loss of CDX2 is an adverse prognostic biomarker in stage IV colorectal cancer. (A) Distribution and dichotomization of CDX2 protein expression according to Allred scores (left) and gene expression (right). (B) Representative images (0.6 mm, captured at 400× magnification) of CDX2‐positive and CDX2‐negative tumors, illustrating specific CDX2 staining in epithelial cells, predominantly in the nuclear compartment, but many cases also showed staining in the cytosol. Scale bar is 0.1 mm. (C) Kaplan–Meier plots showing association between CDX2 expression and survival for cancer stages I–IV separately, based on dichotomized CDX2 expression in the pooled Norwegian series. The log‐rank test was used to test for differences in survival between CDX2‐negative and CDX2‐positive cases, while univariable Cox regression (Wald) was used to generate hazard ratios (HR) and 95% confidence intervals (CI). Relapse was defined only after complete resection. Hence, overall survival was used to evaluate survival in stage IV.

The gene expression values of *CDX2* were dichotomized in the Norwegian series 2 using the Binarization Across Multiple Scales (BASC) algorithm implemented in the R library BiTrinA (Mussel *et al*., 2016) (Fig. 2A). This algorithm calculates a step function in the sorted gene expression data and identifies the threshold value as the location with the strongest discontinuity. *CDX2* gene expression was scored as negative in 50/403 (12.4%) of the cases.

In the Norwegian series 1 analyzed by immunohistochemistry (Fig. 2B), cases (one 0.6‐mm core per patient) were visually evaluated and nuclear staining was measured semiquantitatively with regard to the proportion of positive cells and the intensity of staining, according to the method proposed by Allred *et al*. (1998). All cores were scored concomitantly by an experienced pathologist (LD) and two investigators (RA, RB), blinded to clinical data. Each core was given a total score between 0 and 8 based on the sum of a proportion score between 1 and 5 (0 = none, 1 = less than 1%, 2 = 1–10%, 3 = 11–33%, 4 = 34–66%, and 5 = 67–100%) and an intensity score between 0 and 3 (0 = negative, 1 = weak, 2 = intermediate, 3 = strong). Scores were dichotomized for statistical analyses (CDX2‐negative ≤6; CDX2‐positive >6; Fig. 2A), and 71/642 (11.1%) cases were scored as negative for CDX2 expression. This cutoff compares well with the cutoff determined by the BASC algorithm for the *CDX2* gene expression data, thus facilitating pooled analyses of the two datasets.

As for patients, the BASC algorithm was used to dichotomize cell lines based on CDX2 mRNA expression resulting in 14/35 (40%) cell lines classified as CDX2‐negative. Compared to patient cohorts, the panel is enriched for MSI (13/35, 37%) and the CMS4 subtype (12/35, 34%), explaining the comparatively large number of CDX2‐negative cases. As expected, based on primary tumors, cell line CDX2 status was significantly associated with MSI (Fig. S10) and CMS (*P *=* *0.03 and *P *=* *0.002, respectively; Fisher's exact tests). Cell line gene expression principal component analysis was performed using the R *prcomp* function with the 1000 genes with the largest 10–90% interpercentile range in signal intensities. Gene set analysis was performed using the *camera* function in the R Bioconductor package *limma* (Ritchie *et al*., 2015; Wu and Smyth, 2012). Seventy gene sets were preselected based on relevance for CRC. Cell lines were CMS‐classified as described in Sveen *et al*. (2017).

To evaluate the representativeness of our model system, we performed camera gene set analysis for cell lines and primary tumors separately (Fig. S10). Both CDX2‐negative cell lines and primary tumors showed relative upregulation of TGF‐β‐induced genes and an epithelial–mesenchymal signature. Correspondingly, CDX2‐positive cell lines were characterized by higher expression of gastrointestinal markers and HNF4A target genes. Gene sets discordant between the cell lines and patient samples, such as inflammatory response and IL‐6/JAK/STAT3 signaling, are at least partly attributable to the lack of stromal and immune cell types in cell cultures.

### 2.5. Literature search

We searched the PubMed database from 1966 to January 2018 for prognostic and clinicopathological studies on CDX2 using the MeSH terms ‘CDX2’ and ‘Colorectal neoplasm’ and identified 18 relevant studies (Table S4). Only studies performed on human CRC tissue were included.

### 2.6. *In vitro* drug screen

#### 2.6.1. Cell lines

Cell lines were sourced from commercial vendors and collaborators, and their identities were verified by short tandem repeat profiling using the AmpFℓSTR Identifiler PCR Amplification Kit (Life Technologies by Thermo Fisher Scientific, Waltham, MA, USA). Cell lines were maintained in DMEM/F12 (except from CaCo2 and WiDr cells which were maintained in EMEM) supplemented with fetal bovine serum (FBS), 2 mm glutamine, 100 units·mL^−1^ penicillin, and 100 µg·mL^−1^ streptomycin (Gibco, Life Technologies, Carlsbad, CA, USA) at 37^°^C and 5% CO_2_ in a humidified incubator. All cell lines were enriched with 10% FBS, except for CaCo2 cells, which were enriched with 20% FBS. Cell lines were regularly tested for mycoplasma contamination according to the MycoAlert Mycoplasma Detection Assay (Lonza Cologne AG). Gene expression data were generated using Human Transcriptome Arrays 2.0 (Affymetrix Inc.). Further details are given in Berg *et al*. (Berg *et al*., 2017).

#### 2.6.2. Drug sensitivity and resistance testing

Drug sensitivity and resistance testing (DSRT) was performed using an established high‐throughput platform (Pemovska *et al*., 2013). Cell lines were screened with a library of 461 clinical, emerging, and experimental small‐molecule drugs at five different concentrations over a 10 000‐fold concentration range, including 69 conventional chemotherapeutics analyzed in this study. Prior to DSRT, growth patterns and rates were evaluated by viability assays and microscopy to ensure logarithmic cell growth during the 72 h of drug exposure. Drugs were preprinted on 384‐well plates using liquid acoustic dispensing technology (Echo 550; Labcyte Inc., Sunnyvale, CA, USA). The cells were seeded onto these plates using a Multidrop Combi Reagent Dispenser (Thermo Fisher Scientific) and assessed for viability after 72 h using the CellTiter‐Glo (CTG) assay (Promega, Fitchburg, WI, USA). CTG provides a metabolic readout by producing a luminescent signal proportional to the amount of ATP in the well and thus the number of live cells before cell lysis. Luminescence was measured on a PHERAstar FS microplate reader (BMG Labtech GmbH, Ortenberg, Germany). For each drug, there was one signal value for each of five different concentrations. Drug readouts were compared and normalized to wells with only DMSO (0.1%, negative control) and benzethonium chloride (100 μm, positive control). Drug efficacy was estimated as a drug sensitivity score (DSS) according to the model proposed by Yadav *et al*. (2014). Prior to testing for subtype associations, 24/69 conventional chemotherapeutics were filtered out due to low cross‐sample variance (either having high or no effect across all cell lines).

### 2.7. Statistical analyses

All survival and correlation analyses were performed using spss version 21.0 (IBM Corporation, Armonk, NY, USA). The Kaplan–Meier method was used to generate 5‐year relapse‐free (RFS) and overall survival (OS) plots. Survival curves were compared using the log‐rank test. The generalized Wilcoxon test (Gehan–Breslow) was used in cases where the proportional hazards assumption was violated. Hazard ratios (HR) and confidence intervals (CI) for disease recurrence were calculated using the Cox proportional hazards model. RFS was defined as the time from surgery to the first event of either locoregional recurrence or metastasis, or death from the same cancer, other cancer, non‐cancer‐related death, or death due to treatment. The second primary for the same or other cancer was ignored. Importantly, relapse was defined only after complete resection. Therefore, OS was used to evaluate survival in stage IV and for analyses including stages I–IV, where death from any cause was the only event. All patients were followed up throughout the study period. Evaluated parameters in multivariable models were determined based on clinical relevance and known association with CDX2 expression, including age and gender as background covariates. The independent value of CDX2 was confirmed for all combinations of the covariates included in the final model. Only significant parameters were included in the multivariable model restricted to patients with stage IV disease to optimize the robustness of the model due to lower sample size (stepwise variable selection procedure using forward selection (entry criterion 0.05) and backward elimination (selection stay criterion 0.05)). Patients with missing data were not included in the analyses. Formal interaction tests were integrated in the COX models to assess whether effects were different between subgroups, but must be interpreted carefully due to the low power of such tests. The proportional hazards assumptions were evaluated graphically by plots of log (‐log survival time) versus log time. Subgroup analyses according to tumor stage, MSI status, *BRAF*‐mutation status, tumor location, histopathological grade, and adjuvant treatment were performed based on *a priori* knowledge that these parameters are associated with expression of CDX2, and correction for multiple testing was therefore not performed.

Two‐sided Wilcoxon rank‐sum tests were performed using the R functions *wilcox.test* and *p.adjust* with Benjamini–Hochberg false discovery rate (FDR) estimation adjustment (Benjamini and Hochberg, 1995). Heatmaps were prepared in R. Input was mean‐centered drug sensitivity scores.

For drug sensitivity validation analyses, gene expression data and natural‐log‐transformed IC50 drug sensitivity values were retrieved from http://www.cancerrxgene.org/downloads (Iorio *et al*., 2016) (accessed on April 3, 2017).

*P*‐values and correlation coefficients (*r*) for CDX2 expression in patient samples were generated using Wilcoxon rank‐sum test (exact) when comparing to age, tumor stage, and histopathological grade, and using Fisher exact test when comparing to gender, MSI status, *BRAF*‐mutation status, and tumor location. Correlations were calculated using dichotomized CDX2 expression values. A *P*‐value alpha level of 0.05 (two‐tailed) was considered statistically significant. Exceptions are delineated in the text.

## 3. Results

### 3.1. CDX2 expression has prognostic value in stage IV CRC

A literature review identified 18 studies reporting on loss of CDX2 expression in relation to clinicopathological characteristics and prognosis in CRC (Table S4), of which eleven reported that loss of CDX2 expression was associated with a poor prognosis and three studies reported no difference, while none found that loss of CDX2 was associated with a good prognosis.

Loss of CDX2 expression was found in 50/403 (12.4%) and 71/642 (11.1%) of the samples in the two Norwegian datasets, respectively (totally 11.6% of 1045 patients), and shown to be strongly correlated with low histopathological grade, right‐sided tumors, MSI, *BRAF* mutation, and the undifferentiated CMS1/4 subtypes (Table S2). Univariable survival analyses for stages I–IV showed that loss of CDX2 was significantly associated with a shorter 5‐year OS (log‐rank *P *=* *0.016, *n *=* *1045; Fig. S2A and Table 1), and multivariable analyses showed that CDX2 had prognostic value independent of relevant prognostic factors, including histopathological grade, tumor location, MSI, and *BRAF* mutation (HR 1.53; 95% CI 1.07–2.18; *P *=* *0.021; Table 1 (upper panel)).

**Table 1:** CDX2 is an independent prognostic biomarker in colorectal cancer. Univariable and multivariable survival analyses of CDX2 expression. The Cox proportional hazards regression method (Wald) was used to evaluate univariable and multivariable relationships for CDX2 and clinicopathological and molecular parameters. Abbreviations: G1, high differentiation; G2, moderate differentiation; G3, poor differentiation; MSI, microsatellite‐instable; MSS, microsatellite‐stable; ND, not determined; OS, overall survival

Parameter	Patients, *n* (%)	Univariable analysis (OS)		Multivariable analysis (OS)	
Norwegian series		Hazard ratio (95% CI)	P	Hazard ratio (95%CI)	P
**Stages I‐IV**	**1330 (100)**				
CDX2	1030 (100)				
Positive	924 (88)	1		1	
Negative	121 (12)	1.40 (1.06–1.83)	0.016	1.53 (1.07–2.18)	0.021
ND	285				
Age^a^	1330 (100)	1.03 (1.02–1.03)	2.2 × 10^−11^	1.04 (1.03–1.05)	4.2 × 10^−11^
Gender	1330 (100)				
Female	694 (52)	1		1	
Male	636 (48)	0.98 (0.83–1.15)	0.77	1.14 (0.92–1.41)	0.24
Tumor stage					
I	223 (17)	1		1	
II	535 (40)	1.75 (1.27–2.42)		1.21 (0.81–1.80)	
III	356 (27)	2.88 (2.08–3.99)		2.94 (1.99–4.34)	
IV	212 (16)	11.8 (8.51–16.3)	2.9 × 10^−90^	12.5 (8.20–19.0)	7.1 × 10^−54^
ND	4				
Histopathological grade					
G1	95 (7)	1		1	
G2	1020 (80)	1.26 (0.89–1.77)		1.23 (0.79–1.92)	
G3	165 (13)	2.03 (1.37–2.99)	3.8 × 10^−5^	2.25 (1.33–3.82)	4.0 × 10^−4^
Mucinous*	16				
ND	34				
MSI status					
MSS	1036 (84)	1		1	
MSI	200 (16)	0.70 (0.54–0.89)	0.0047	0.37 (0.24–0.58)	1.7 × 10^−5^
ND	94				
Tumor location					
Proximal colon	539 (41)	1		1	
Distal colon	420 (32)	1.05 (0.87–1.27)		0.99 (0.77–1.28)	
Rectum	342 (26)	0.78 (0.63–0.96)		0.91 (0.68–1.22)	
Synchronous	26 (2)	0.66 (0.35–1.24)	0.023	0.65 (0.27–1.61)	0.75
BRAF					
Wt	988 (84)	1		1	
Mut	188 (16)	1.19 (0.94–1.49)	0.14	1.31 (0.90‐1.92)	0.16
ND	154				
Chemotherapy					
Yes	213 (16)	1		1	
No	1088 (84)	1.62 (1.32–1.98)	3.1 × 10^−6^	0.70 (0.51–0.95)	0.022
ND	29				
Patient series					
Norwegian series 1	927 (70)	1		1	
Norwegian series 2	403 (30)	0.57 (0.46–0.69)	1.3 × 10^−8^	0.52 (0.42–0.65)	1.4 × 10^−8^
**Stage IV** b	**212 (100)**				
CDX2					
Positive	139 (85)	1		1	
Negative	25 (15)	3.92 (2.47–6.22)	6.4 × 10^−9^	2.38 (1.26–4.48)	0.0074
ND	48				
Histopathological grade					
G1 + G2	163 (80)	1		1	
G3	41 (20)	2.93 (2.04–4.22)	7.1 × 10^−9^	2.14 (1.22–3.76)	0.0080
Mucinous*	4				
ND	4				
Chemotherapy					
No	92 (49)	1		1	
Yes	95 (51)	0.64 (0.47–0.87)	0.0039	0.60 (0.42–0.86)	0.0053
ND	25				
Patient series					
Norwegian series 1	159 (75)	1		1	
Norwegian series 2	53 (25)	0.54 (0.38–0.75)	3.5 × 10^−4^	0.61 (0.42–0.90)	0.012

a Hazard ratios are given per year of age. NDs and samples indicated with an asterisk were excluded from the statistical analyses.

b Minimal model including only significant variables (stepwise selection). G1 and G2 were grouped due to low number of G1 cases in stage IV.

However, loss of CDX2 expression was weakly correlated with advanced cancer stage (Table S2), and by analyzing the four stages separately, a significant association with a worse 5‐year overall survival was found only in stage IV [HR 3.96; 95% CI 2.50–6.28; *P *=* *3.1 × 10^−10^, *n *=* *164; formal test for interaction (full multivariable model, stages I–III versus stage IV: *P *=* *0.024; Figs 2C and S2BC)] and confirmed in multivariable analysis restricted to stage IV [HR 2.38; 95% CI 1.26–4.48; *P *=* *0.0074; Table 1 (lower panel)].

### 3.2. CDX2 expression is associated with response to chemotherapy

**Figure 3:**
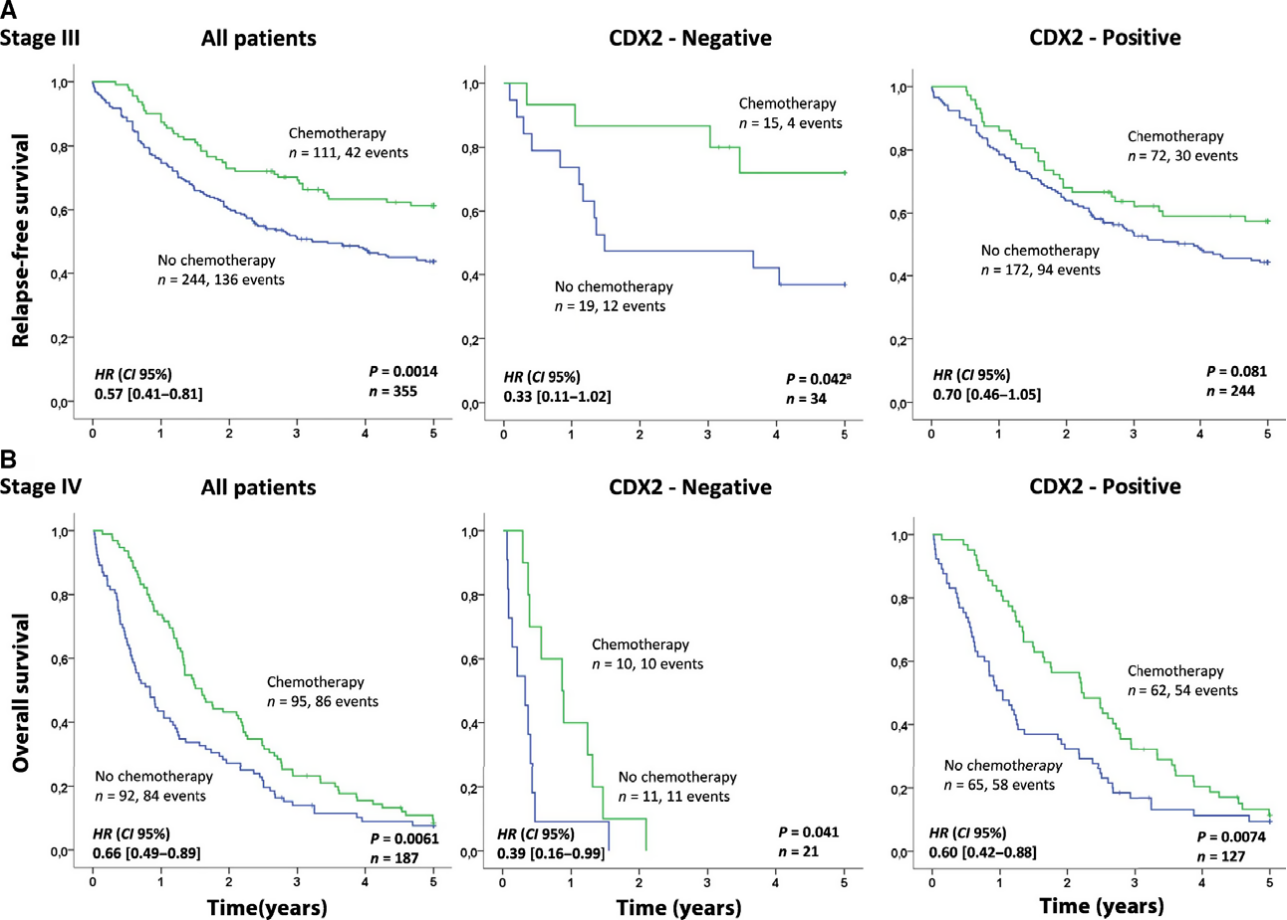
Association between chemotherapy and survival related to CDX2 expression for stage III patients (A) and for stage IV patients (B). The Kaplan–Meier method was used to generate the survival plots and the log‐rank test was used to test for differences in survival between CDX2‐negative and CDX2‐positive cases, while univariable Cox regression (Wald) was used to generate hazard ratios (HR) and 95% confidence intervals (CI). Relapse was defined only after complete resection; hence, overall survival was used to evaluate survival in stage IV. ^a^The proportional hazards assumption is violated and the *P*‐value was generated using the generalized Wilcoxon test (Gehan–Breslow). Here, both the log‐rank test and the Wilcoxon test provide identical results.

Of the patients with stage III disease, the 32% who received adjuvant chemotherapy had better outcome than those who did not (5‐year RFS, 61% versus 44%: *P *=* *0.0014, *n *=* *355; Fig. 3A (left panel)). The difference in the 5‐year RFS rate between treated and untreated patients was larger for patients with CDX2‐negative tumors (75% versus 37%, respectively: *P *=* *0.042, *n *=* *34; Fig. 3A (middle panel) and Fig. S3) compared to patients with CDX2‐positive tumors (58% *versus* 46%, respectively: *P *=* *0.081, *n *=* *244; Fig. 3A (right panel) and Fig. S3), although the number of samples and events were too small to detect an interaction effect (formal test for interaction: *P *=* *0.61). Interestingly, this apparent benefit from adjuvant chemotherapy in patients with loss of CDX2 expression was found in both the MSI and microsatellite‐stable (MSS) subgroups separately (Fig. S4). Notably, the lack of a prognostic effect of CDX2 in stage III (shown in Fig. 2) may be confounded by treatment with adjuvant chemotherapy; a weak association with survival was observed for patients who did not receive adjuvant chemotherapy (Fig. S5A). However, in stage IV, the prognostic value of CDX2 expression was independent of chemotherapy (Fig. S5B), and CDX2 was not found to have a predictive value for treatment response in this cancer stage, as the rate of 5‐year OS was similar for patients with CDX2‐negative and CDX2‐positive tumors when comparing those who received chemotherapy to patients who did not receive chemotherapy (Fig. 3B; formal test for interaction: *P *=* *0.31).

**Figure 4:**
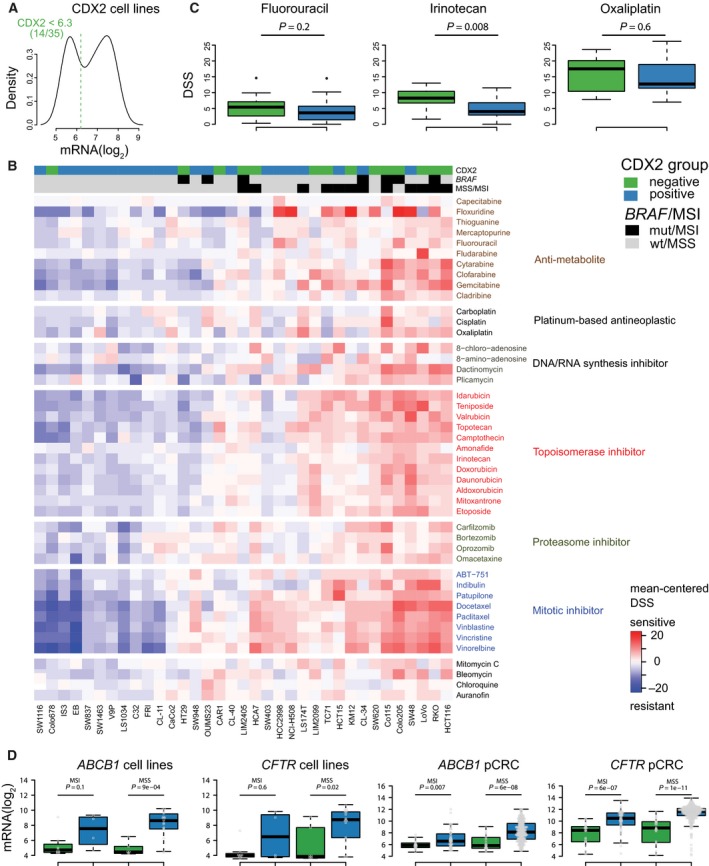
CDX2‐negative CRC cell lines are more sensitive to conventional chemotherapeutics. CDX2‐negative cell lines are indicated by green, while CDX2‐positive cell lines are indicated by blue. (A) Cell lines were dichotomized according to *CDX2* mRNA expression, as shown in the density plot. Dashed vertical line indicates threshold value, as determined by the Binarization Across Multiple Scales algorithm. (B) Drug responses of 35 CRC cell lines to conventional chemotherapeutics. Values represented are mean‐centered drug sensitivity scores with red indicating higher relative sensitivity. Samples are ordered according to complete linkage agglomerative clustering of the pairwise Manhattan distance matrix based on global gene expression. Drugs are ordered according to the mechanism of action. (C) Boxplots show DSS values for commonly used chemotherapeutics for CRC treatment. Higher values indicate higher drug sensitivity. (D) Boxplots show that both *CDX2*‐negative cell lines and primary CRCs have significantly reduced mRNA expression of *ABCB1*/*MDR1* and *CFTR*/*ABCC7*. *P*‐values are from two‐sided Wilcoxon rank‐sum tests. Abbreviations: DSS, drug sensitivity score; GSA, gene set analysis; MSI, microsatellite‐instable; MSS, microsatellite‐stable; pCRC, primary colorectal cancer.

We hypothesized that loss of CDX2 expression in tumors confers increased sensitivity to chemotherapeutic drugs, and investigated this possibility *in vitro* by high‐throughput drug screening and gene expression analyses of 35 CRC cell lines, where 14/35 (40%) were CDX2‐negative (Fig. 4A). The cell line dataset was representative of the primary tumors with respect to CDX2 expression, based on molecular associations (MSI and CMS) and gene set analyses (additional details in Methods and Supporting Information Fig. S10).

Sixty‐nine conventional chemotherapeutics were included in the drug screen (45 drugs showed differential responses between the cell lines and were used in the analyses). Comparison of drug responses showed stronger overall sensitivity among CDX2‐negative compared to CDX2‐positive cell lines (Fig. 4B). Thirty of the drugs showed a significant difference in response, and none were more effective in CDX2‐positive cell lines (Wilcoxon rank‐sum test with FDR adjustment, *P *<* *0.1; Table S5). Specifically, the standard CRC chemotherapeutic drug irinotecan was more effective in CDX2‐negative cell lines (Wilcoxon rank‐sum test, *P *=* *0.008 (with FDR adjustment, *P *=* *0.02); Fig. 4C), while the differences observed for fluorouracil and oxaliplatin were not statistically significant. Notably, irinotecan is a prodrug that is activated in the liver, and this result should be interpreted with caution. However, several other topoisomerase inhibitors were also significantly more effective in CDX2‐negative cell lines as compared to CDX2‐positive cell lines (Table S5). Considering only MSS cell lines (*n *=* *23), 41/45 drugs had higher average response in the CDX2‐negative cell lines (Fig. S6), demonstrating that this effect was not determined by MSI status.

To validate this pharmacogenomic relationship, we took advantage of a large dataset of gene expression and drug sensitivity profiles (with missing values) for 46 CRC cell lines (24 nonoverlapping) published by Iorio *et al*. (Iorio *et al*., 2016). Based on mRNA expression, 16/46 (35%) cell lines were scored as CDX2‐negative. Considering 18 chemotherapeutic drugs, 17/18 had on average lower IC50 values (higher efficacy) in CDX2‐negative cell lines, with a statistically significant association for eight drugs (Wilcoxon rank‐sum test with FDR adjustment, *P *<* *0.1; Table S6). When considering only the 24 cell lines not overlapping with our in‐house dataset, likewise, 17/18 had on average lower IC50 values in CDX2‐negative cell lines, although none were statistically significant following FDR adjustment (Table S7).

The multidrug resistance genes *MDR1* (also called *ABCB1*) and *CFTR* (also called *ABCC7*), coding for two ATP‐dependent drug efflux pumps, are transcriptional downstream targets of CDX2 (Kerschner and Harris, 2012; Koh *et al*., 2016; Takakura *et al*., 2010; Yan *et al*., 2015). We found that both *MDR1/ABCB1* and *CFTR/ABCC7* were significantly downregulated in CDX2‐negative patient tumors and cell lines, independent of MSI status (Fig. 4D).

### 3.3. Loss of CDX2 expression identifies a poor prognostic subgroup among patients with stages I–III and *BRAF* mutations

**Figure 5:**
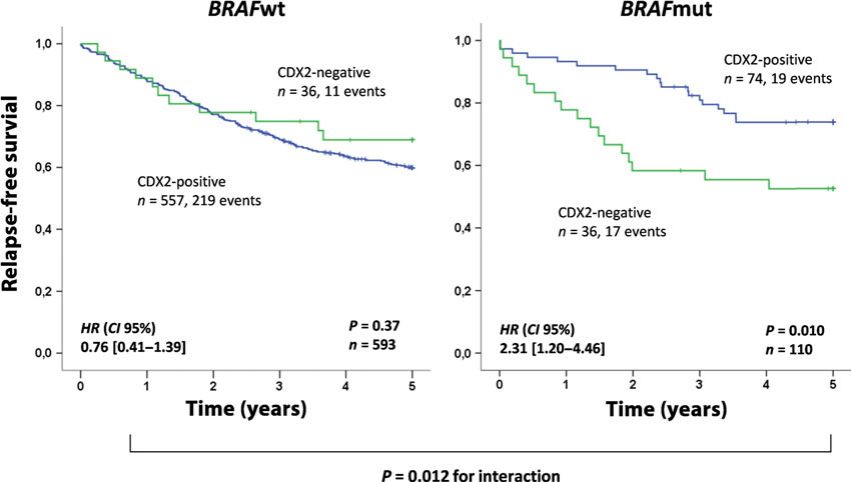
Loss of CDX2 expression identifies a poor prognostic subgroup among patients with stages I–III and *BRAF* mutation. Prognostic value of CDX2 expression in relation to *BRAF* status in stage I–III chemo‐naïve patients. The Kaplan–Meier method was used to generate the survival plots and the log‐rank test was used to test for differences in survival between CDX2‐negative and CDX2‐positive cases, while univariable Cox regression (Wald) was used to generate hazard ratios (HR) and 95% confidence intervals (CI).

Due to the strong prognostic effect of CDX2 loss in stage IV and the apparent association between CDX2 expression and benefit from adjuvant chemotherapy in stage III, we subsequently performed combined prognostic biomarker analyses for untreated stage I–III patients. The prognostic value of CDX2 was independent of MSI status (Fig. S7A) and CMS (Fig. S7B). Loss of CDX2 expression was associated with shorter patient survival both in CMS1 (5‐year OS: 75% versus 61%; HR 1.98; CI, 0.78–5.03; *P *=* *0.14, *n *=* *63) and in CMS4 separately (5‐year OS: 52% versus 30%; HR 1.64; CI, 0.75–3.58; *P *=* *0.21, *n *=* *62; Fig. 5B), while the prevalence in CMS2/3 was too low for prognostic evaluation. However, CDX2 showed strong prognostic value specifically among patients with *BRAF*‐mutated tumors (*P *=* *0.012 for the interaction; Fig. 5), and this association was independent of the MSI status (Figs S8 and S9).

## 4. Discussion

CDX2 is an emerging biomarker in CRC, but for optimal interpretation of its true prognostic and predictive value, it is important to define the most appropriate context and patient subgroups. The present study shows that loss of CDX2 expression has a negative prognostic impact in stage IV CRC. This was demonstrated in two unselected, Norwegian population‐based patient series and was independent of clinicopathological and molecular parameters known to be associated with CDX2, as well as chemotherapy. Our data and the literature review suggest that CDX2 is not a sufficiently reliable biomarker to identify patients with a high risk of relapse after surgical treatment for stage II and stage III CRC. Few studies have reported the stage‐specific prognostic value of CDX2. Of note, Bae *et al*. (2015) and Zhang *et al*. (2017) reported particular prognostic value of CDX2 for stage IV cancers, in accordance with our findings. Dalerba *et al*. (2016) reported prognostic value for CDX2 in stages II–IV, but we could not confirm the prognostic impact of CDX2 in stages II–III. Dalerba and colleagues used one pooled discovery dataset (*n *=* *466) comprising four different selected patient series (http://www.ncbi.nlm.nih.gov/geo/query/acc.cgi?acc=GSE14333, http://www.ncbi.nlm.nih.gov/geo/query/acc.cgi?acc=GSE17538, http://www.ncbi.nlm.nih.gov/geo/query/acc.cgi?acc=GSE31595, and http://www.ncbi.nlm.nih.gov/geo/query/acc.cgi?acc=GSE37892) and a smaller selected validation dataset (NCI‐CDP, *n *=* *314). There may be a selection bias in this pooled series in relation to clinicopathological data that can explain this discrepancy. Furthermore, our unselected population‐based series include elderly patients, which may also provide some explanation for this contrasting finding. Of potential clinical relevance, we show that patients with stage IV CRC and CDX2‐negative tumors have a median survival of 6.7 months as compared to 23.5 months for patients with CDX2‐positive tumors, suggesting that loss of CDX2 can be a useful biomarker to identify patients with limited benefit from surgery.

Our clinical data are in line with the proposed predictive value of CDX2 for adjuvant chemotherapy in stage III CRC (Dalerba *et al*., 2016) and show that loss of CDX2 appears to be associated with a higher 5‐year RFS rate after chemotherapy, independent of MSI status. However, due to small sample sizes and the challenges to evaluate benefit from adjuvant treatment, these findings should be interpreted with caution. Notably, there was no indication of an association between CDX2 expression and benefit from chemotherapy in stage IV CRC. Accordingly, the proposed predictive value of CDX2 may be context‐dependent; however, it is difficult to compare adjuvant treatment for stage III with largely palliative treatment for stage IV, and many other factors are likely to have an impact on the survival of patients with stage IV CRC.

Thus, to investigate the potential pharmacogenomic association between loss of CDX2 expression and sensitivity to chemotherapy, we analyzed drug sensitivity in preclinical models. Several studies show that CRC cell lines recapitulate the main molecular phenotypes observed in primary CRC (Ahmed *et al*., 2013; Barretina *et al*., 2012; Berg *et al*., 2017; Medico *et al*., 2015; Mouradov *et al*., 2014), which substantiate their value as preclinical model systems to assess a variety of pharmacogenomic relationships. Consistent with the observed clinical benefit in patients with CDX2‐negative tumors in stage III, loss of CDX2 expression was strongly associated with sensitivity to conventional chemotherapeutics *in vitro*, both in our drug screen dataset and in a large public dataset, again independent of MSI status. This coincided with the significant downregulation of the two multidrug resistance genes and downstream CDX2 targets *MDR1/ABCB1* and *CFTR/ABCC7* in CDX2‐negative cell lines. This was further validated in patient tumors, providing a potential mechanism explaining why loss of CDX2 appears to promote response to chemotherapy.

In Norway, most patients with stage II disease at the time of diagnosis are not offered adjuvant chemotherapy, thus precluding analysis of a hypothesized predictive value of CDX2 expression in this patient group. Furthermore, for stage III, the number of CDX2‐negative cases and events were not sufficient to test reliably for interaction between CDX2 expression and treatment with adjuvant chemotherapy. It should be noted that interaction tests performed for some subgroup analyses were limited by sample size, hence underpowered to reliably detect significant differences between groups. Adjuvant treatment for stage III (<75 years of age) became standard in Norway in 1997, explaining the relatively low frequency of patients receiving adjuvant chemotherapy in our patient series (patient inclusion from 1993).

The relationship between CDX2 loss and chemotherapy in a population‐based cohort is limited by several confounders not captured in the survival models such as performance status. However, our finding agrees well with the predictive value of CDX2 in stage III reported in Dalerba *et al*. (2016), and the proposed relationship between CDX2 expression and drug response is supported by comprehensive pharmacogenomic assessments in independent datasets. In our patient series, the frequency of loss of CDX2 expression was in line with published data, and we confirm well‐known associations with clinicopathological and molecular parameters, which support the representativeness of our series. Although combined analyses of CDX2 expression at the gene and protein levels might introduce unintended bias, gene and protein expression levels of CDX2 have been shown to be strongly correlated (Olsen *et al*., 2016), an observation we confirm in cell lines (Fig. S1C). All analyses performed for the separate datasets were highly comparable, altogether providing a sound rationale for combined analyses of the different data types in relation to clinical outcomes.

The molecular parameters MSI status, *BRAF* mutations, and CMS are all associated with prognosis in CRC. To our knowledge, no studies have assessed the impact of all biomarkers on the prognostic value of CDX2. We show that CDX2 retains prognostic value in a multivariable model including these important covariates. Furthermore, explorative analyses within the chemo‐naïve stage I–III subgroup suggest that CDX2 carries prognostic information for cancers with *BRAF* mutations, within both the MSI and MSS subtypes separately, highlighting CDX2 as a potential biomarker with additional prognostic information to MSI and *BRAF* status. This finding is in line with recent studies reporting synergistic oncogenic activity between loss of CDX2 and *BRAF* mutation in serrated tumors (Sakamoto *et al*., 2017; Tong *et al*., 2017), which is associated with more aggressive disease and a poor prognosis (Garcia‐Solano *et al*., 2010).

The recent definition of four CMS groups has provided a rational framework to refine classification and stratification of CRC (Guinney *et al*., 2015). In our datasets, we observe strong correlation between loss of CDX2 and the CMS1 and CMS4 subtypes, in accordance with the strong association of CDX2 loss with MSI (CMS1) and worse prognosis (CMS4), and loss of differentiation, a hallmark of CDX2 loss in cancer (Suh *et al*., 1994). In contrast to a recent study (Pilati *et al*., 2017), we showed that loss of CDX2 is associated with shorter survival in both CMS1 and CMS4. This suggests that CDX2 has prognostic value across CMSs, although CMS2 and CMS3 tumors rarely show loss of CDX2.

CDX2 is an independent prognostic biomarker in CRC, but the prognostic value is limited to stage IV cancers. Pharmacogenomic analyses of preclinical CRC models show that CDX2‐negative cells are more sensitive to conventional chemotherapeutics and show significant downregulation of genes conferring multidrug resistance.

## Authors’ contributions

JB, AS, LD, AN, RA, and RAL conceived and designed the study. All authors acquired biological and clinical data. JB, AS, RB, PWE, MK, MG, AN, LD, RA, and RAL analyzed and interpreted the data. JB drafted the manuscript, and all authors were involved in revision of the manuscript and have read and approved the final version. RAL supervised the study.

## Supporting information


**Table S1.** REMARK checklist.
**Table S2.** Clinicopathological data for all patients included in the study.
**Table S3.** Comparisons of patient and tumor characteristics for evaluable versus nonevaluable CDX2 protein expression in the Norwegian series 1.
**Table S4.** Literature review on CDX2 as a biomarker in colorectal cancer.
**Table S5.** Conventional chemotherapeutic drugs with significant differential drug sensitivity according to CDX2 expression in cell lines.
**Table S6.** Validation of differential drug sensitivity to conventional chemotherapeutics according to CDX2 expression in cell lines.
**Table S7.** Validation of differential drug sensitivity to conventional chemotherapeutics according to CDX2 expression in cell lines (only nonoverlapping cell lines).
**Fig. S1.** Correlation between CDX2 gene expression and CDX2 protein expression in CRC cell lines.
**Fig. S2.** Association between CDX2 expression and prognosis.
**Fig. S3.** Association between CDX2 expression and adjuvant chemotherapy for stage III CRC in the two Norwegian series 1 and 2, separately.
**Fig. S4.** Association between CDX2 expression and adjuvant chemotherapy according to microsatellite instability status in stage III CRC patients.
**Fig. S5.** The prognostic value of CDX2 according to chemotherapy in A) stage III and B) stage IV CRC.
**Fig. S6.** Association between CDX2 expression and response to conventional chemotherapeutics in microsatellite‐stable (MSS) cell lines.
**Fig. S7.** Prognostic value of CDX2 expression according to microsatellite instability status for stage I–III chemo‐naïve patients (A) and CMS (B, stages I–IV).
**Fig. S8.** Prognostic value of CDX2 expression according to *BRAF*‐mutation status (stage I–III chemo‐naïve) for patients with MSI (A) and MSS (B).
**Fig. S9.** Prognostic associations between CDX2 expression and *BRAF*‐mutation status according to microsatellite instability status for stage I–III chemo‐naïve patients.
**Fig. S10.** Representativeness of CRC cell line panel related to CDX2 expression and microsatellite instability status.
