# Supplementary material for: Prognostic, predictive, and pharmacogenomic assessments of CDX2 refine stratification of colorectal cancer
Source: Mol Oncol. 2018 Aug 15;12(9):1639–55. doi: 10.1002/1878-0261.12347 (PMC6120232; doi:10.1002/1878-0261.12347)
Supplement: Supplementary file 1 — Table S1. REMARK checklist. Table S2. Clinicopathological data for all patients included in the study. Table S3. Comparisons of patient and tumor characteristics for evaluable versus nonevaluable CDX2 protein expression in the Norwegian series 1. Table S4. Literature review on CDX2 as a biomarker in colorectal cancer. Table S5. Conventional chemotherapeutic drugs with significant differential drug sensitivity according to CDX2 expression in cell lines. Table S6. Validation of differential drug sensitivity to conventional chemotherapeutics according to CDX2 expression in cell lines. Table S7. Validation of differential drug sensitivity to conventional chemotherapeutics according to CDX2 expression in cell lines (only nonoverlapping cell lines). Fig. S1. Correlation between CDX2 gene expression and CDX2 protein expression in CRC cell lines. Fig. S2. Association between CDX2 expression and prognosis. Fig. S3. Association between CDX2 expression and adjuvant chemotherapy for stage III CRC in the two Norwegian series 1 and 2, separately. Fig. S4. Association between CDX2 expression and adjuvant chemotherapy according to microsatellite instability status in stage III CRC patients. Fig. S5. The prognostic value of CDX2 according to chemotherapy in A) stage III and B) stage IV CRC. Fig. S6. Association between CDX2 expression and response to conventional chemotherapeutics in microsatellite‐stable (MSS) cell lines. Fig. S7. Prognostic value of CDX2 expression according to microsatellite instability status for stage I–III chemo‐naïve patients (A) and CMS (B, stages I–IV). Fig. S8. Prognostic value of CDX2 expression according to BRAF‐mutation status (stage I–III chemo‐naïve) for patients with MSI (A) and MSS (B). Fig. S9. Prognostic associations between CDX2 expression and BRAF‐mutation status according to microsatellite instability status for stage I–III chemo‐naïve patients. Fig. S10. Representativeness of CRC cell line panel related to CDX2 expression and microsatellite in [file MOL2-12-1639-s001.docx]

Prognostic, predictive and pharmacogenomic assessments of CDX2 refine stratification of colorectal cancer

# Supplementary Tables and Figures

**Table S1. REMARK checklist.**

| **Item to be reported** | | **Page no.** |
| --- | --- | --- |
| **INTRODUCTION** | |  |
| 1 | State the marker examined, the study objectives, and any pre-specified hypotheses. | 1, 4, 5 |
| **MATERIALS AND METHODS** | |  |
| *Patients* | |  |
| 2 | Describe the characteristics (e.g., disease stage or co-morbidities) of the study patients, including their source and inclusion and exclusion criteria. | 6,16,27 |
| 3 | Describe treatments received and how chosen (e.g., randomized or rule-based). | 9, 11, 27 |
| *Specimen characteristics* | |  |
| 4 | Describe type of biological material used (including control samples) and methods of preservation and storage. | 6 |
| *Assay methods* | |  |
| 5 | Specify the assay method used and provide (or reference) a detailed protocol, including specific reagents or kits used, quality control procedures, reproducibility assessments, quantitation methods, and scoring and reporting protocols. Specify whether and how assays were performed blinded to the study endpoint. | 6-7, 24-25 |
| *Study design* | |  |
| 6 | State the method of case selection, including whether prospective or retrospective and whether stratification or matching (e.g., by stage of disease or age) was used. Specify the time period from which cases were taken, the end of the follow-up period, and the median follow-up time. | 6 |
| 7 | Precisely define all clinical endpoints examined. | 7 |
| 8 | List all candidate variables initially examined or considered for inclusion in models. | 7-8 |
| 9 | Give rationale for sample size; if the study was designed to detect a specified effect size, give the target power and effect size. | 6, 24 |
| *Statistical analysis methods* | |  |
| 10 | Specify all statistical methods, including details of any variable selection procedures and other model-building issues, how model assumptions were verified, and how missing data were handled. | 6-8, 24-25 |
| 11 | Clarify how marker values were handled in the analyses; if relevant, describe methods used for cutpoint determination. | 6-7 |
| **RESULTS** | |  |
| *Data* | |  |
| 12 | Describe the flow of patients through the study, including the number of patients included in each stage of the analysis (a diagram may be helpful) and reasons for dropout. Specifically, both overall and for each subgroup extensively examined report the numbers of patients and the number of events. | 16 |
| 13 | Report distributions of basic demographic characteristics (at least age and sex), standard (disease-specific) prognostic variables, and tumor marker, including numbers of missing values. | 16, 27-28 |
| *Analysis and presentation* | |  |
| 14 | Show the relation of the marker to standard prognostic variables. | 19, 27 |
| 15 | Present univariable analyses showing the relation between the marker and outcome, with the estimated effect (e.g., hazard ratio and survival probability). Preferably provide similar analyses for all other variables being analyzed. For the effect of a tumor marker on a time-to-event outcome, a Kaplan-Meier plot is recommended. | 9, 18-19 |
| 16 | For key multivariable analyses, report estimated effects (e.g., hazard ratio) with confidence intervals for the marker and, at least for the final model, all other variables in the model. | 19 |
| 17 | Among reported results, provide estimated effects with confidence intervals from an analysis in which the marker and standard prognostic variables are included, regardless of their statistical significance. | 8, 19 |
| 18 | If done, report results of further investigations, such as checking assumptions, sensitivity analyses, and internal validation. | 8 |
| **DISCUSSION** | |  |
| 19 | Interpret the results in the context of the pre-specified hypotheses and other relevant studies; include a discussion of limitations of the study. | 11-12 |
| 20 | Discuss implications for future research and clinical value. | 11-12 |

**Table S2. Clinicopathological data for all patients included in the study.** *P*-values indicate correlation between CDX2 expression and the indicated patient characteristic. Wilcoxon rank-sum test (exact) was used to correlate CDX2 expression to age, stage and grade, while the Fisher’s exact test was used to correlate CDX2 expression to gender, MSI, *BRAF* status and tumor location. NDs and samples indicated with an asterisk were excluded from the statistical analyses. Abbreviations: G1, high differentiation; G2, moderate differentiation; G3, poor differentiation; MSI, microsatellite instable; MSS, microsatellite stable; ND, not determined.

**
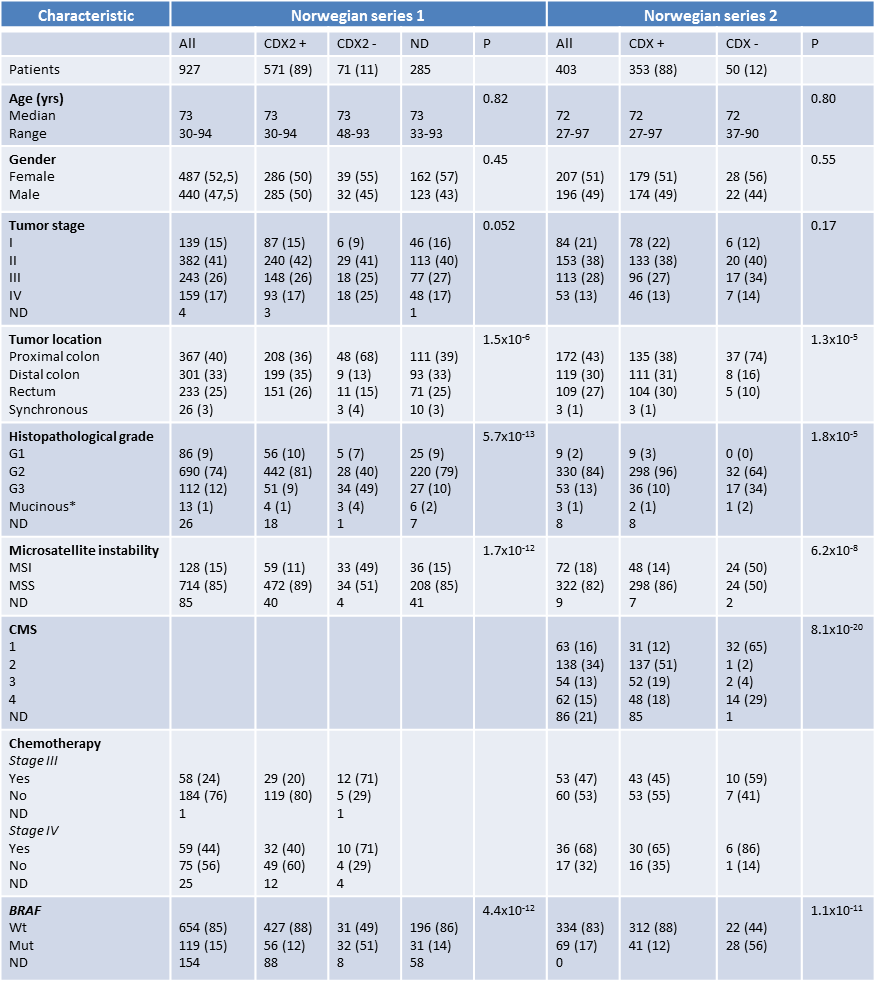
**

**Table S3. Comparisons of patient and tumor characteristics for evaluable *versus* non-evaluable CDX2 protein expression in the Norwegian series 1.**

**
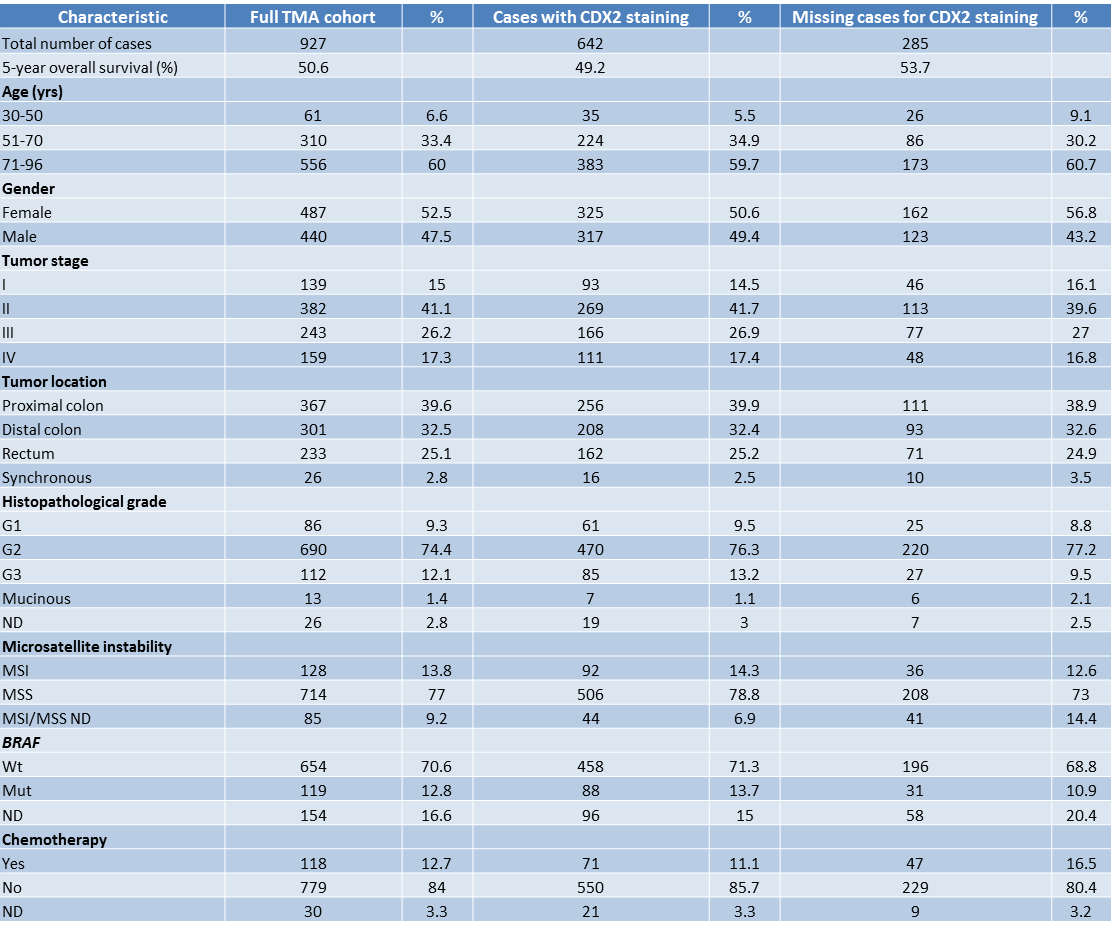
**

**Table S4. Literature review on CDX2 as a biomarker in colorectal cancer (CRC).** The PubMed database from 1966 to January 2018 was searched for prognostic and clinicopathological studies on CDX2 in CRC. The search was limited to studies performed on human CRC tissue. Abbreviations: HR, hazard ratio; CI, confidence interval; pAb, polyclonal antibody; WTS, whole tissue section; IHC, immunohistochemistry; GEA, gene expression analysis; MSI-H, microsatellite instability – high; CMS, consensus molecular subtype; DFS, disease-free survival; DSS, disease-specific survival; RFS, relapse-free survival; CSS, cancer-specific survival; OS, overall survival; n.s., not significant; Pts, patients; DIA, digital image analysis (continuous scaling; investigators evaluated thresholds of positivity with regard to tumor cell percentages and staining intensity levels); * Study without prognostic data; Study showing no prognostic value of CDX2; **•** Study showing that loss of CDX2 is significantly associated with poor prognosis.

**
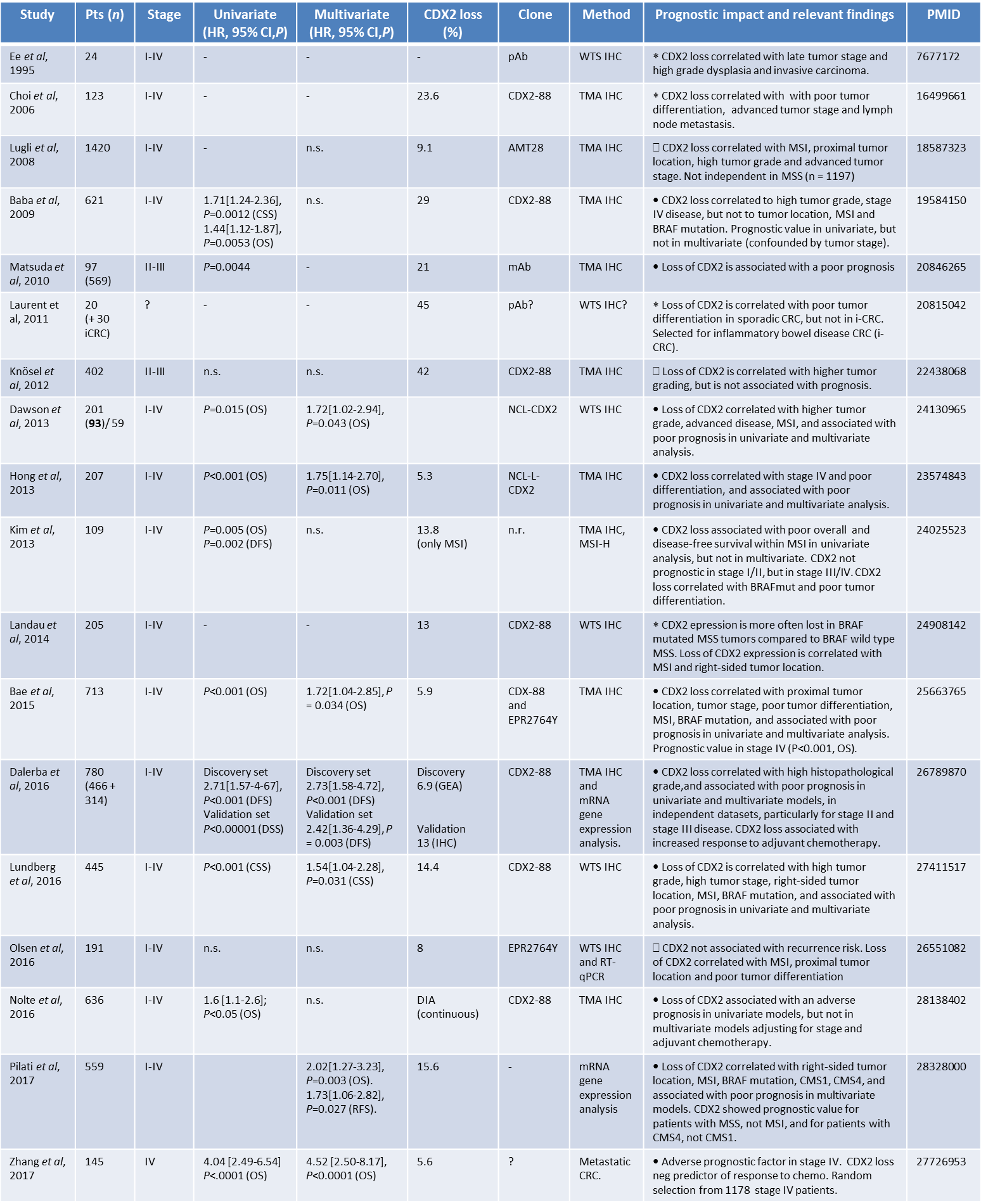
**

**Figure S1. Correlation between *CDX2* gene expression and CDX2 protein expression in CRC cell lines.** A cell line micro array including 35 CRC cell lines (from 29 unique patients) was constructed (1.0 mm cores) and 4 µm thick sections were immunohistochemically stained with a rabbit monoclonal antibody against the CDX2 protein (Clone EPR2764Y, Cell Marque, Rocklin, CA, U.S.A.) and imaged at 0.33 µm/pixel resolution using Pannoramic P250 Flash II whole slide scanner (3DHistech, Budapest, Hungary) equipped with Zeiss Plan-Apochromat 20x objective (NA 0.8). Images were exported as JPEG2000 format (95 % quality). The CDX2 antibody has previously been shown to provide very similar detection of CDX2 as the CDX2-88 clone (Bae et al, WJB 2015; PMID: 25663765). For CDX2 protein expression analysis, the Hematoxylin-DAB images were color deconvoluted using IHC Profiler tool (Varghese et al. 2014). Cell nuclei segmentation and CDX2 intensity measurement were done using CellProfiler 2.1.2 software platform (Kamentsky et al. 1979; PMID: 391998), and the mean CDX2 channel intensity was measured within expanded nuclei (1-pixel expansion) in each spot. A) CDX2-positive cell line. B) CDX2-negative cell line. C) Scatterplot showing correspondence between CDX2 gene and protein expression in the 29 CRC cell lines. *r* is the Pearson correlation coefficient. Samples are colored according to CDX2 status as determined from *CDX2* mRNA. Scale bar is 0.1 mm.

**
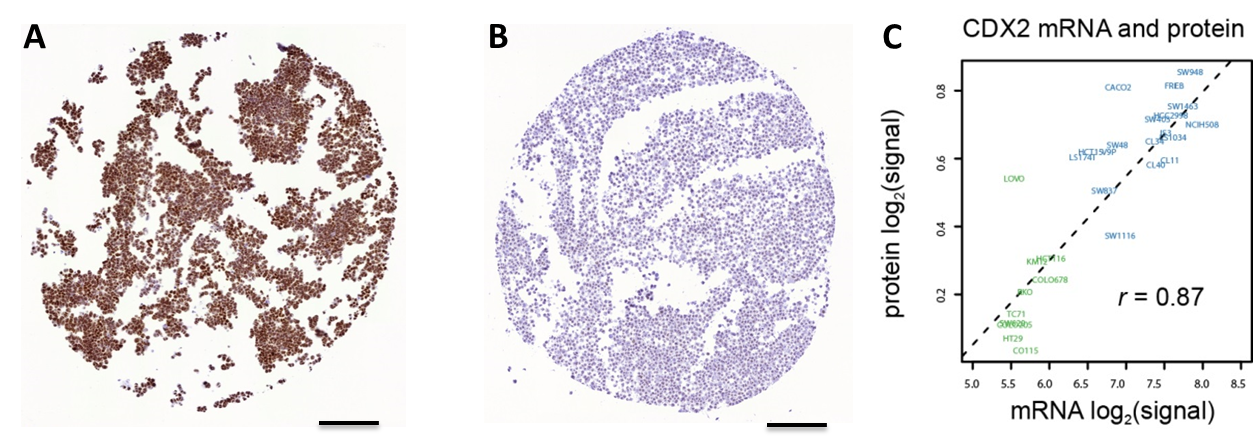
**

**Figure S2. Association between CDX2 expression and prognosis.** All patients (stage I-IV) (A), Norwegian series 1 by tumor stage (B), and Norwegian series 2 by tumor stage (C). The Kaplan-Meier method was used to generate the survival plots and the log rank test was used to test for differences in survival between CDX2-negative and CDX2-positive cases, while univariate Cox regression (Wald) was used to generate hazard ratios (HR) and 95% confidence intervals (CI). Relapse was defined only after complete resection. Hence, overall survival was used to evaluate survival in stage IV. ^a^The proportional hazards assumption is violated and the *P*-value was generated using the Generalized Wilcoxon test (Gehan-Breslow).

**
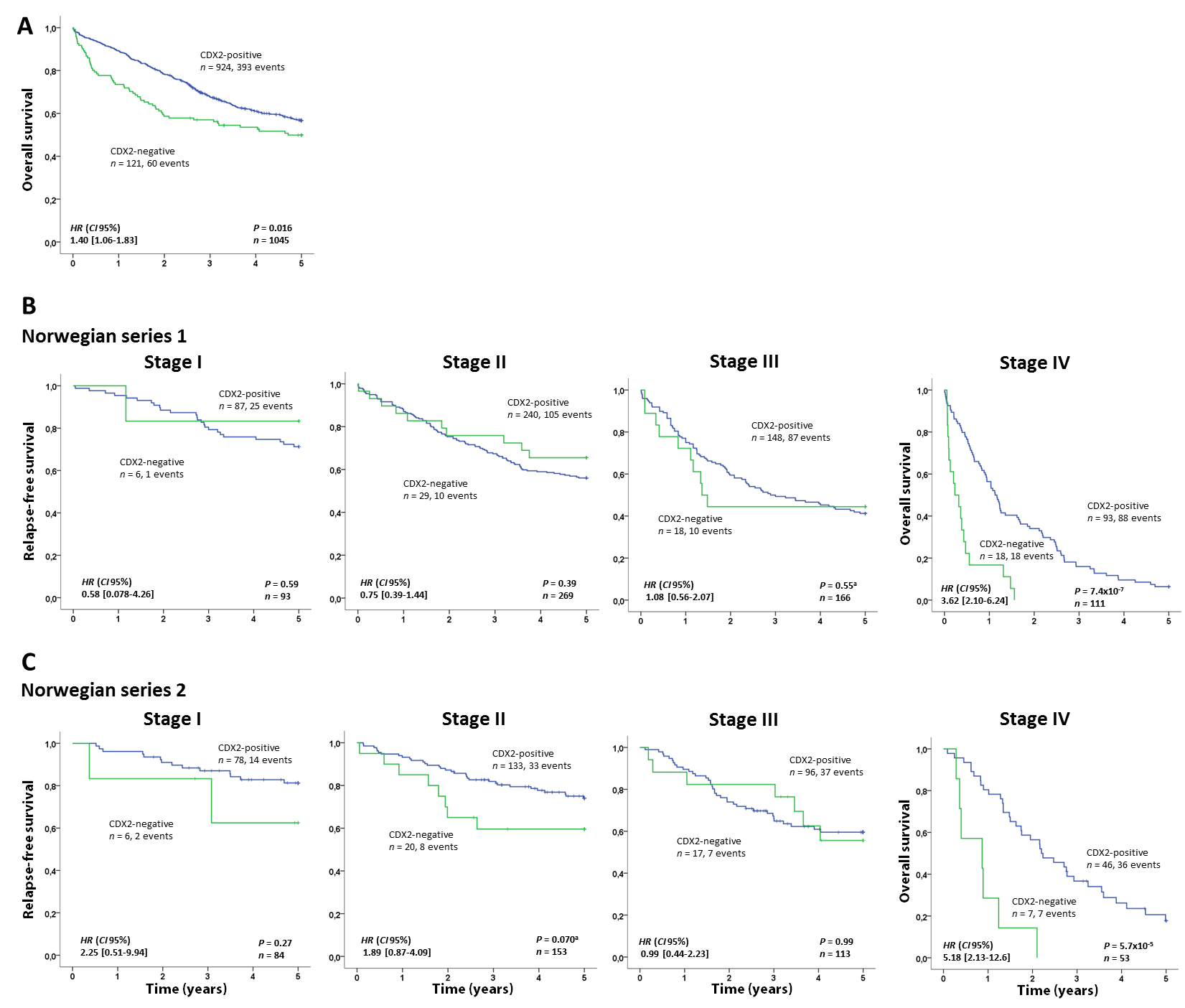
**

**Figure S3. Association between CDX2 expression and adjuvant chemotherapy for stage III CRC in the two Norwegian series 1 and 2, separately.** The Kaplan-Meier method was used to generate the survival plots and the log rank test was used to test for differences in survival between CDX2-negative and CDX2-positive cases, while univariate Cox regression (Wald) was used to generate hazard ratios (HR) and 95% confidence intervals (CI).

**
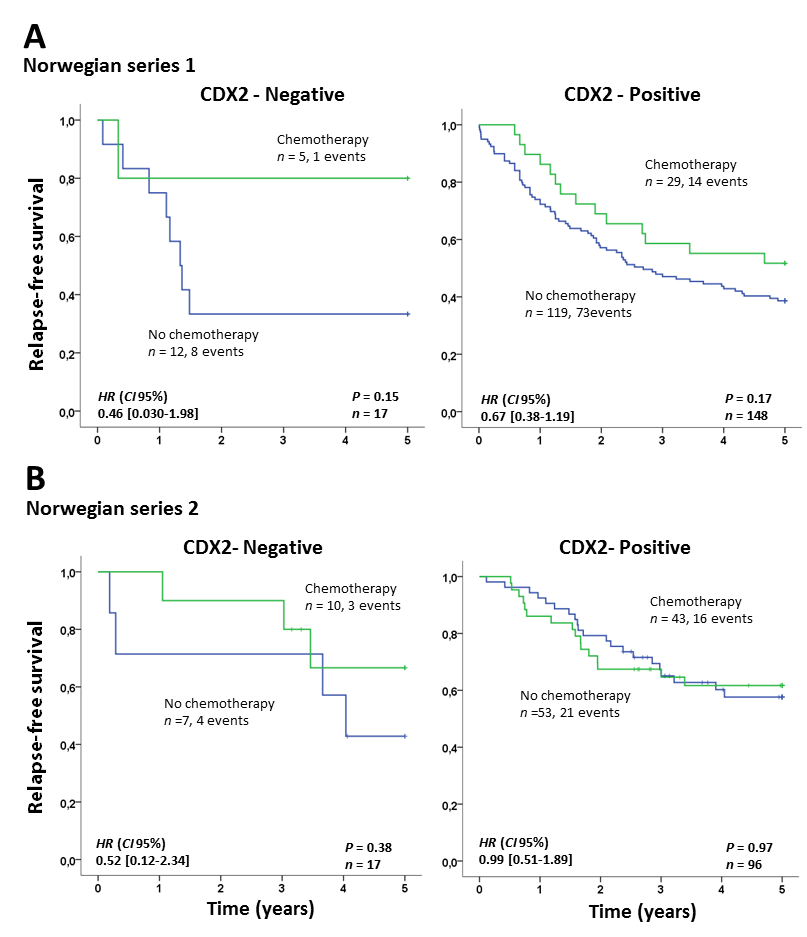
**

**Figure S4. Association between CDX2 expression and adjuvant chemotherapy according to microsatellite instability status in stage III CRC patients.** The Kaplan-Meier method was used to generate the survival plots and the log rank test was used to test for differences in survival between CDX2-negative and CDX2-positive cases, while univariate Cox regression (Wald) was used to generate hazard ratios (HR) and 95% confidence intervals (CI). ^a^The proportional hazards assumption is violated and the *P*-value was generated using the Generalized Wilcoxon test (Gehan-Breslow).

**
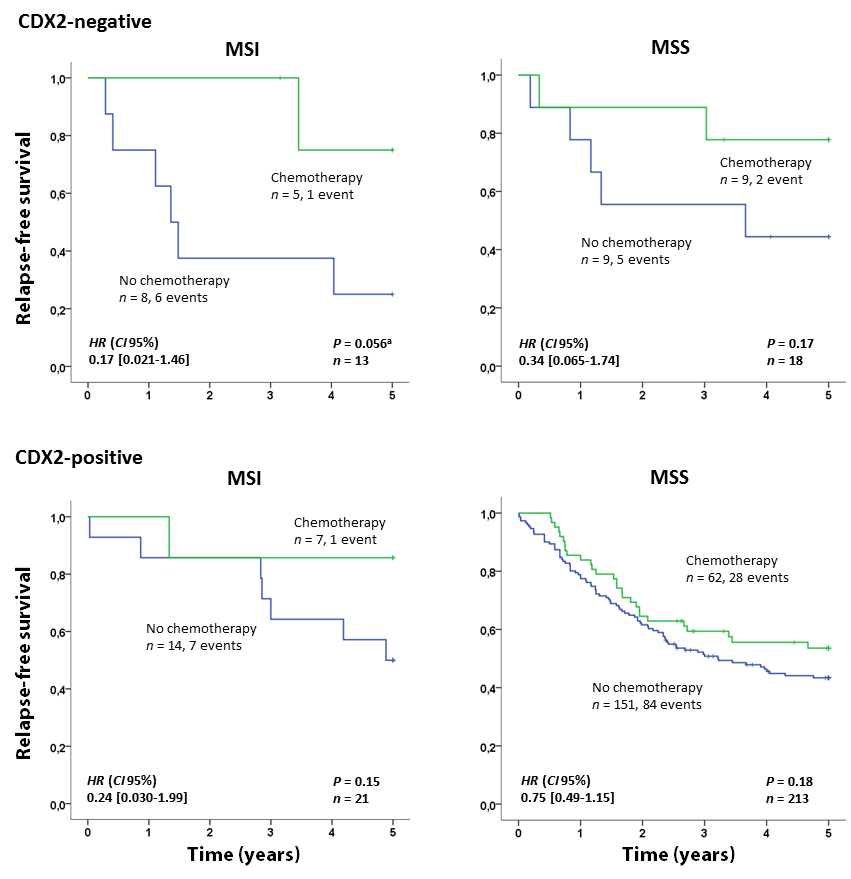
**

**Figure S5. The prognostic value of CDX2 according to chemotherapy in A) stage III and B) stage IV CRC.** The Kaplan-Meier method was used to generate the survival plots and the log rank test was used to test for differences in survival between CDX2-negative and CDX2-positive cases, while univariate Cox regression (Wald) was used to generate hazard ratios (HR) and 95% confidence intervals (CI). Relapse was defined only after complete resection. Hence, overall survival was used to evaluate survival in stage IV. ^a^The proportional hazards assumption is violated and the *P*-value was generated using the Generalized Wilcoxon test (Gehan-Breslow).

**
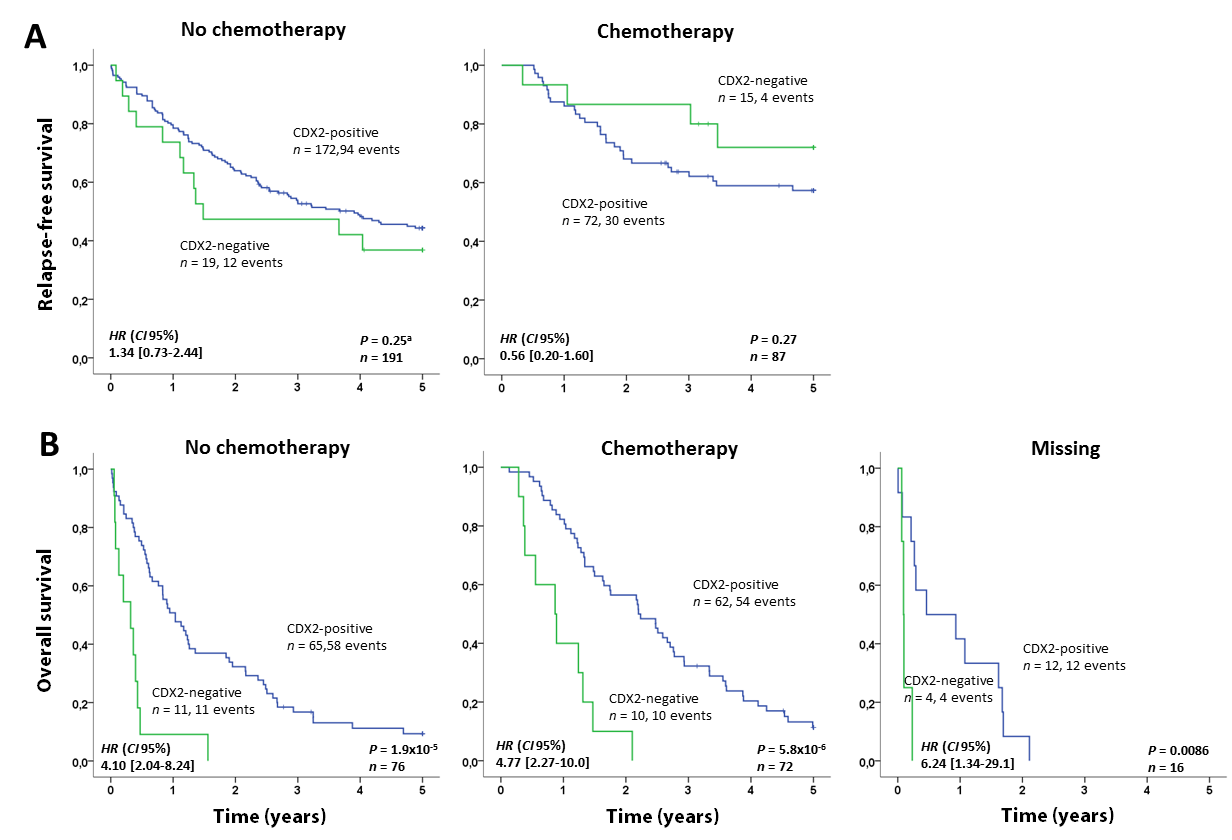
**

**Figure S6. Association between CDX2 expression and response to conventional chemotherapeutics in microsatellite stable (MSS) cell lines.** Values represented are mean-centered drug sensitivity scores with red indicating higher relative sensitivity. Samples are ordered according to complete linkage agglomerative clustering of the pair-wise Manhattan distance matrix.

**
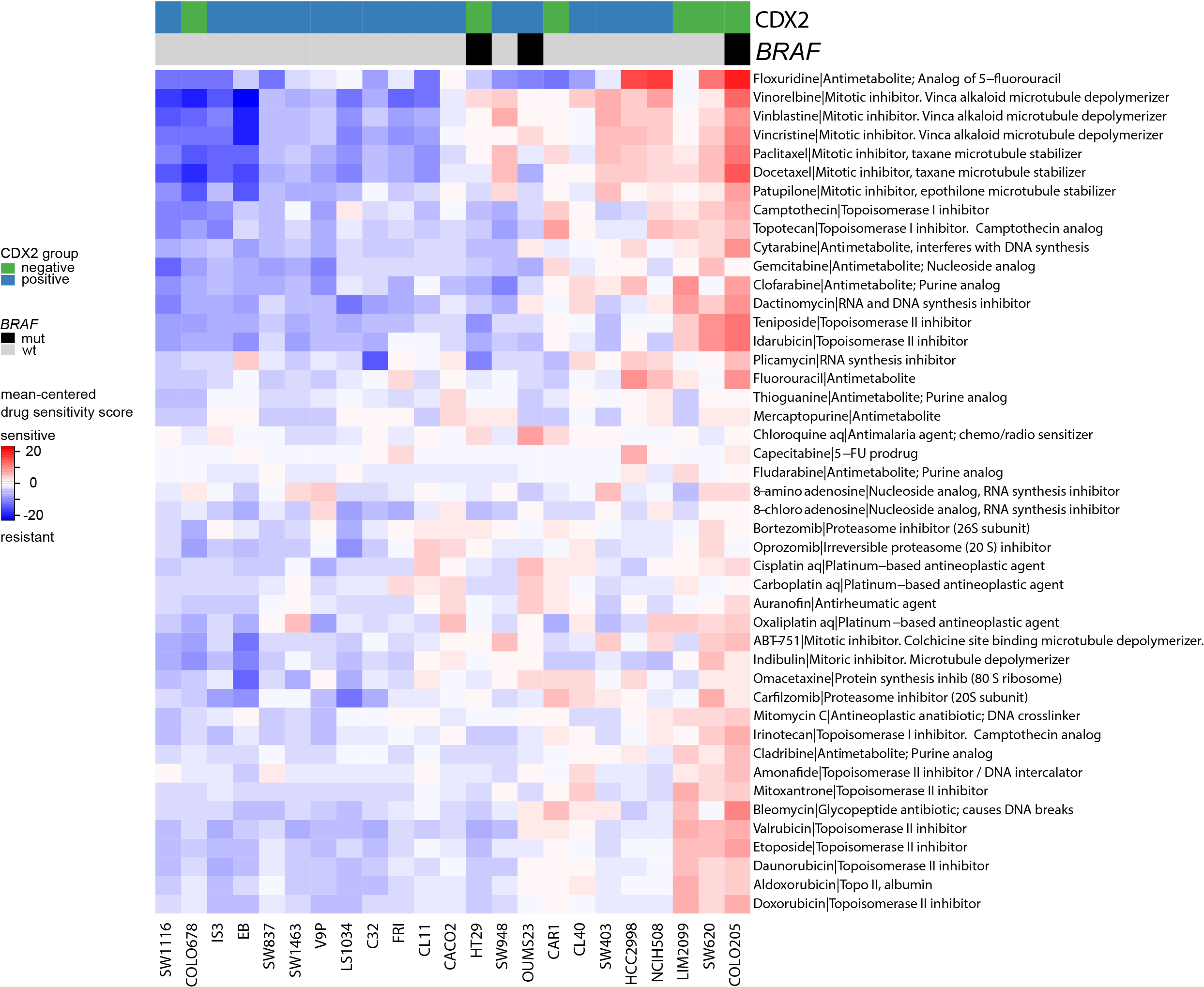
**

**Table S5. Conventional chemotherapeutic drugs with significant differential drug sensitivity according to *CDX2* expression in cell lines.** Positive DSS-values indicate higher activity in *CDX2*-negative cell lines. Wilcoxon rank-sum test was used to test differences in drug response. Abbreviations: FDR, false discovery rate; DSS, drug sensitivity score. ^a^Prodrug, activated in the liver.

| **Drug** | **Mean difference**  **DSS** | ***P*-value** | **FDR** | **Mechanism of action** |
| --- | --- | --- | --- | --- |
| Carfilzomib | 5.55 | 0.001 | 0.015 | Proteasome inhibitor (20S subunit) |
| Idarubicin | 6.91 | 0.001 | 0.015 | Topoisomerase II inhibitor |
| Dactinomycin | 7.09 | 0.001 | 0.015 | RNA and DNA synthesis inhibitor |
| Doxorubicin | 4.15 | 0.002 | 0.015 | Topoisomerase II inhibitor |
| Topotecan | 5.74 | 0.002 | 0.015 | Topoisomerase I inhibitor. Camptothecin analog |
| Etoposide | 4.40 | 0.002 | 0.015 | Topoisomerase II inhibitor |
| Clofarabine | 6.15 | 0.003 | 0.015 | Anti-metabolite; Purine analog |
| Teniposide | 6.40 | 0.003 | 0.015 | Topoisomerase II inhibitor |
| Vincristine | 7.52 | 0.004 | 0.015 | Mitotic inhibitor |
| Bleomycin | 4.16 | 0.004 | 0.015 | Glycopeptide antibiotic; causes DNA breaks |
| Gemcitabine | 6.62 | 0.004 | 0.015 | Antimetabolite; Nucleoside analog |
| Irinotecan^a^ | 3.37 | 0.004 | 0.015 | Topoisomerase I inhibitor. Camptothecin analog |
| Daunorubicin | 4.10 | 0.005 | 0.015 | Topoisomerase II inhibitor |
| Valrubicin | 5.16 | 0.005 | 0.015 | Topoisomerase II inhibitor |
| Paclitaxel | 6.94 | 0.005 | 0.015 | Mitotic inhibitor, taxane microtubule stabilizer |
| Camptothecin | 5.04 | 0.006 | 0.018 | Topoisomerase I inhibitor |
| Mitoxantrone | 2.92 | 0.008 | 0.021 | Topoisomerase II inhibitor |
| Docetaxel | 7.50 | 0.011 | 0.025 | Mitotic inhibitor, taxane microtubule stabilizer |
| Vinblastine | 6.47 | 0.011 | 0.025 | Mitotic inhibitor. |
| Cytarabine | 4.74 | 0.011 | 0.025 | Anti-metabolite, interferes with DNA synthesis |
| Amonafide | 2.27 | 0.012 | 0.025 | Topoisomerase II inhibitor / DNA intercalator |
| Aldoxorubicin | 3.13 | 0.012 | 0.025 | Topoisomerase II inhibitor |
| Vinorelbine | 8.45 | 0.013 | 0.025 | Mitotic inhibitor |
| Indibulin | 5.18 | 0.014 | 0.025 | Mitoric inhibitor. Microtubule depolymerizer |
| Cladribine | 2.36 | 0.019 | 0.034 | Anti-metabolite; Purine analog |
| Oprozomib | 3.28 | 0.023 | 0.040 | Irreversible proteasome (20 S) inhibitor |
| Fludarabine | 2.44 | 0.026 | 0.044 | Anti-metabolite; Purine analog |
| Auranofin | 1.95 | 0.028 | 0.045 | Anti-rheumatic agent |
| 8-chloro-adenosine | 3.53 | 0.033 | 0.051 | RNA synthesis inhibitor; Nucleoside analog |
| Omacetaxine | 2.75 | 0.059 | 0.089 | Protein synthesis inhibitor (80 S ribosome) |

**Table S6. Validation of differential drug sensitivity to conventional chemotherapeutics according to *CDX2* expression in cell lines.** The IC50-value indicates the effect size of the differential drug sensitivity; a positive value means that the drug is more effective in *CDX2*-negative cell lines. Wilcoxon rank-sum test was used to test differences in drug response. Abbreviations: FDR, false discovery rate. Drug response data are from Iorio *et al*.

| **Drug** | **Median IC50 difference (uM)** | ***P*-value** | **FDR** | **Mechanism of action** |
| --- | --- | --- | --- | --- |
| Vinblastine | 0.088 | 0.0004 | 0.005 | Mitotic inhibitor |
| Docetaxel | 0.029 | 0.0004 | 0.005 | Mitotic inhibitor, taxane microtubule stabilizer |
| Vinorelbine | 0.098 | 0.0024 | 0.013 | Mitotic inhibitor |
| Etoposide | 29 | 0.0027 | 0.013 | Topoisomerase II inhibitor |
| Bleomycin | 37 | 0.0046 | 0.017 | Glycopeptide antibiotic; causes DNA breaks |
| Doxorubicin | 1.2 | 0.013 | 0.042 | Topoisomerase II inhibitor |
| Paclitaxel | 0.15 | 0.022 | 0.06 | Mitotic inhibitor, taxane microtubule stabilizer |
| SN-38 | 0.042 | 0.028 | 0.07 | Topoisomerase I inhibitor |
| Temozolomide | 145 | 0.058 | 0.11 | Alkylating agent |
| Bortezomib | 0.0095 | 0.065 | 0.11 | Proteasome inhibitor (26S subunit) |
| Camptothecin | 0.024 | 0.069 | 0.11 | Topoisomerase I inhibitor |
| Gemcitabine | 0.72 | 0.083 | 0.12 | Antimetabolite; Nucleoside analog |
| Cisplatin | 29 | 0.148 | 0.20 | Platinum-based antineoplastic agent |
| Epothilone B | 0.012 | 0.17 | 0.22 | Microtubule stabiliser |
| Mitomycin-C | 0.68 | 0.21 | 0.25 | Antineoplastic anatibiotic; DNA crosslinker |
| 5-Fluorouracil | 6.5 | 0.54 | 0.61 | Antimetabolite |
| Cytarabine | 0.49 | 0.58 | 0.61 | Antimetabolite |
| Methotrexate | -0.12 | 0.68 | 0.68 | Antimetabolite |

**Table S7. Validation of differential drug sensitivity to conventional chemotherapeutics according to *CDX2* expression in cell lines (only non-overlapping cell lines).** The IC50-value indicates the effect size of the differential drug sensitivity; a positive value means that the drug is more effective in *CDX2*-negative cell lines. Wilcoxon rank-sum test was used to test differences in drug response. Abbreviations: FDR, false discovery rate. Drug response data are from Iorio *et al*.

| **Drug** | **Median IC50 difference (uM)** | ***P*-value** | **FDR** | **Mechanism of action** |
| --- | --- | --- | --- | --- |
| Bleomycin | 60 | 0.015 | 0.28 | Glycopeptide antibiotic; causes DNA breaks |
| Docetaxel | 0.018 | 0.040 | 0.38 | Mitotic inhibitor, taxane microtubule stabilizer |
| SN-38 | 0.043 | 0.10 | 0.41 | Topoisomerase I inhibitor |
| Bortezomib | 0.021 | 0.11 | 0.41 | Proteasome inhibitor (26S subunit) |
| Doxorubicin | 1.4 | 0.13 | 0.41 | Topoisomerase II inhibitor |
| Vinblastine | 0.091 | 0.13 | 0.41 | Mitotic inhibitor |
| Epothilone B | 0.017 | 0.16 | 0.43 | Mitotic inhibitor |
| Gemcitabine | 0.96 | 0.28 | 0.54 | Antimetabolite; Nucleoside analog |
| Etoposide | 4.7 | 0.34 | 0.54 | Topoisomerase II inhibitor |
| Vinorelbine | 0.063 | 0.34 | 0.54 | Mitotic inhibitor |
| Paclitaxel | 0.27 | 0.34 | 0.54 | Mitotic inhibitor |
| 5-Fluorouracil | -11 | 0.39 | 0.57 | Antimetabolite |
| Cisplatin | 9.1 | 0.49 | 0.67 | Platinum-based antineoplastic agent |
| Camptothecin | 0.020 | 0.55 | 0.67 | Topoisomerase I inhibitor |
| Temozolomide | 32 | 0.56 | 0.67 | Alkylating agent |
| Methotrexate | 0.49 | 0.60 | 0.67 | Antimetabolite |
| Mitomycin-C | 0.68 | 0.80 | 0.84 | DNA crosslinker |
| Cytarabine | 0.19 | 0.84 | 0.84 | Antimetabolite |

**Figure S7. Prognostic value of CDX2 expression according to microsatellite instability status for stage I-III chemo naïve patients (A) and CMS (B, stage I-IV).** The Kaplan-Meier method was used to generate the survival plots and the log rank test was used to test for differences in survival between CDX2-negative and CDX2-positive cases, while univariate Cox regression (Wald) was used to generate hazard ratios (HR) and 95% confidence intervals (CI).

**
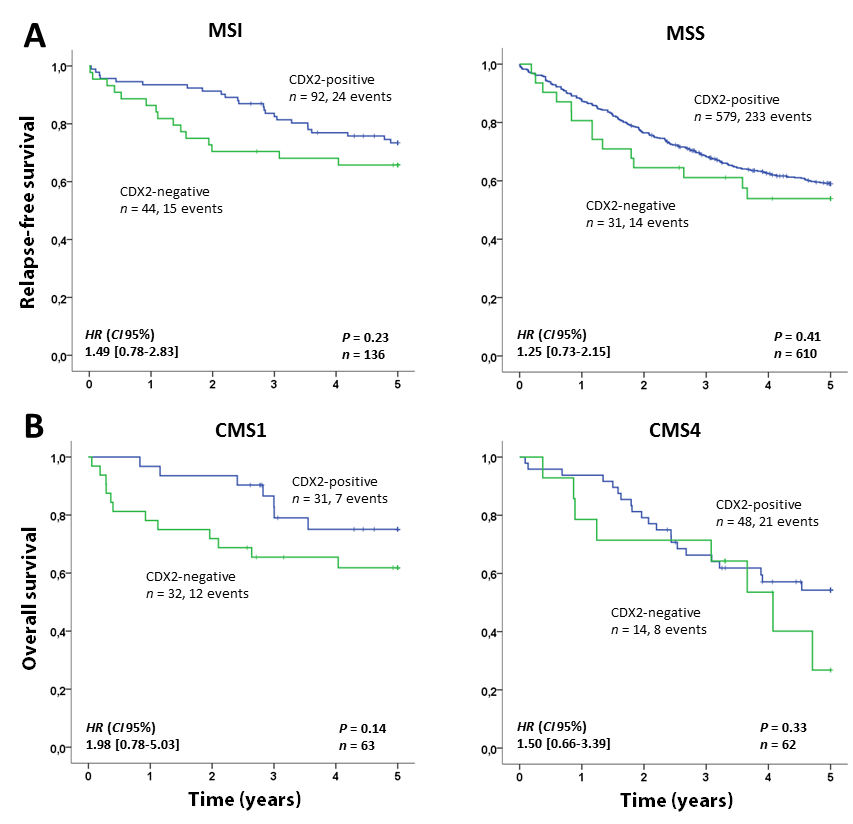
**

**Figure S8. Prognostic value of CDX2 expression according to *BRAF* mutation status (stage I-III chemo naïve) for patients with MSI (A) and MSS (B).** The Kaplan-Meier method was used to generate the survival plots and the log rank test was used to test for differences in survival between CDX2-negative and CDX2-positive cases, while univariate Cox regression (Wald) was used to generate hazard ratios (HR) and 95% confidence intervals (CI).

**
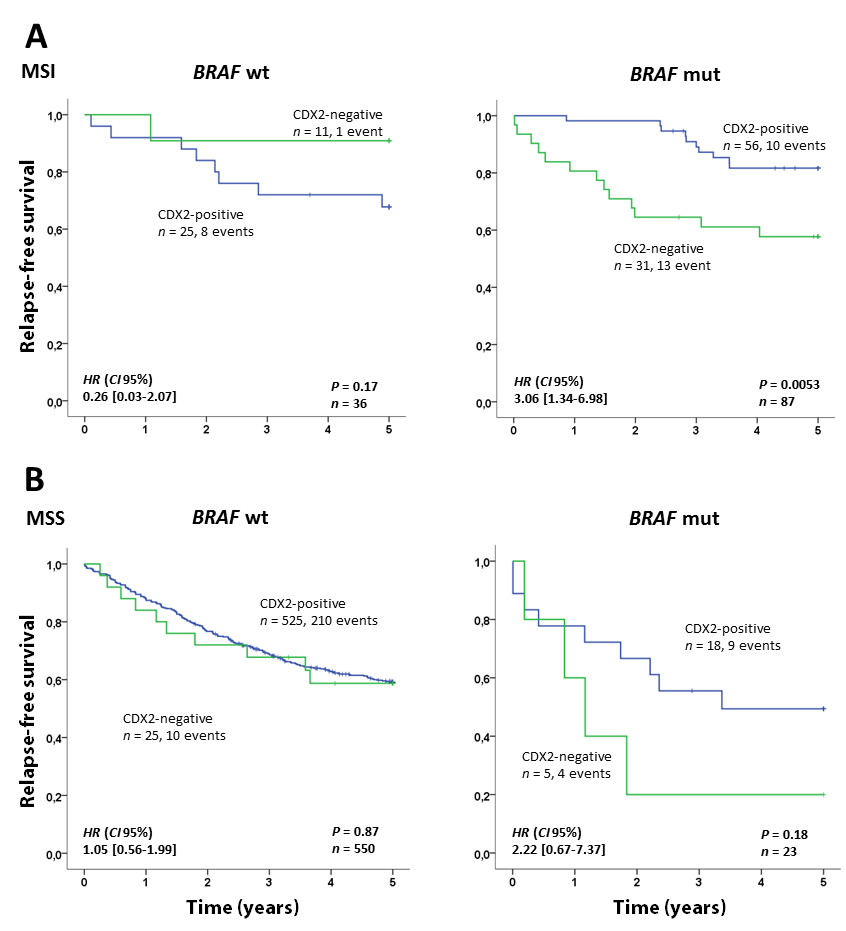
**

**Figure S9. Prognostic associations between CDX2 expression and *BRAF* mutation status according to microsatellite instability status for stage I-III chemo naïve patients.** The Kaplan-Meier method was used to generate the survival plots and the log rank test was used to test for differences in survival between the four groups.

**
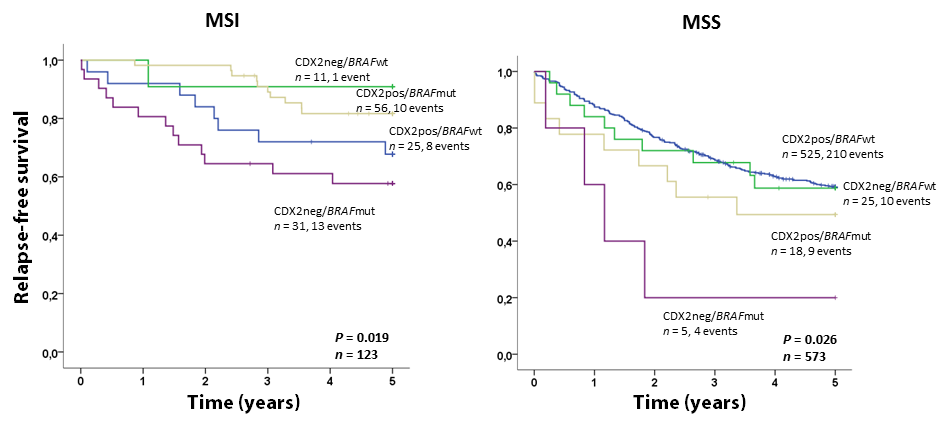
**

**Figure S10. Representativeness of CRC cell line panel related to *CDX2* expression and microsatellite instability status.** A) Unsupervised principal component analysis (PCA) of mRNA expression for 35 CRC cell lines with *CDX2*-positive and -negative cell lines colored blue and green, respectively. Point type indicates whether the cell line is microsatellite instable (MSI, triangle) or microsatellite stable (MSS, circle). Percentages indicate percent of total variance explained by the first two principal components. B) Barplot shows the top-20 most significantly different gene sets from camera gene set analysis (GSA). *CDX2*-negative cell lines and primary CRCs are characterized by relatively lower expression of gastro-intestinal markers, are more MSI-like and have reduced TGFβ-signaling. Gene set details are found in Berg *et al*.

**
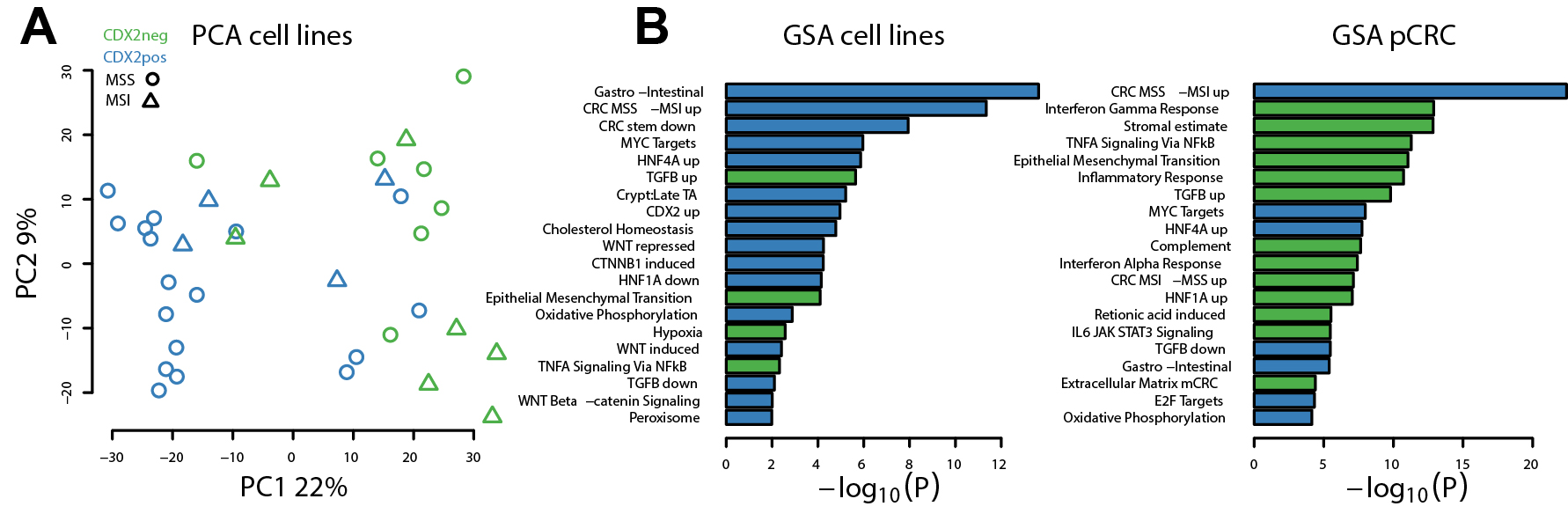
**
